# Chemical profiling of Sanjin tablets and exploration of their effective substances and mechanism in the treatment of urinary tract infections

**DOI:** 10.3389/fchem.2023.1179956

**Published:** 2023-06-20

**Authors:** Meng-Yuan Li, Yang Li, Li-Li Wang, Feng Xu, Xu-Yan Guo, Jing Zhang, Yang Lv, Peng-Pu Wang, Shun-Qi Wang, Jian-Guo Min, Xun Zou, Shao-Qing Cai

**Affiliations:** ^1^ School of Pharmacy, Henan University of Chinese Medicine, Zhengzhou, China; ^2^ School of Pharmaceutical Sciences, Peking University, Beijing, China; ^3^ Guilin Sanjin Pharmaceutical Company Limited, Guilin, China

**Keywords:** Sanjin tablets, urinary tract infections, chemical profiling, LC-MS^n^, network pharmacology, molecular docking

## Abstract

**Introduction:** Sanjin tablets (SJT) are a well-known Chinese patent drug that have been used to treat urinary tract infections (UTIs) for the last 40 years. The drug consists of five herbs, but only 32 compounds have been identified, which hinders the clarification of its effective substances and mechanism.

**Methods:** The chemical constituents of SJT and their effective substances and functional mechanism involved in the treatment of UTIs were investigated by using high performance liquid chromatography-electrospray ionization-ion trap-time of flight-mass spectrometry (HPLC-ESI-IT-TOF-MS^n^), network pharmacology, and molecular docking.

**Results:** A total of 196 compounds of SJT (SJT-MS) were identified, and 44 of them were unequivocally identified by comparison with the reference compounds. Among 196 compounds, 13 were potential new compounds and 183 were known compounds. Among the 183 known compounds, 169 were newly discovered constituents of SJT, and 93 compounds were not reported in the five constituent herbs. Through the network pharmacology method, 119 targets related to UTIs of 183 known compounds were predicted, and 20 core targets were screened out. Based on the “compound–target” relationship analysis, 94 compounds were found to act on the 20 core targets and were therefore regarded as potential effective compounds. According to the literature, 27 of the 183 known compounds were found to possess antimicrobial and anti-inflammatory activities and were verified as effective substances, of which 20 were first discovered in SJT. Twelve of the 27 effective substances overlapped with the 94 potential effective compounds and were determined as key effective substances of SJT. The molecular docking results showed that the 12 key effective substances and 10 selected targets of the core targets have good affinity for each other.

**Discussion:** These results provide a solid foundation for understanding the effective substances and mechanism of SJT.

## 1 Introduction

Urinary tract infections (UTIs) are common infections mainly caused by uropathogenic *Escherichia coli* (UPEC). According to statistics, the incidence of UTIs has been increasing worldwide over the past 30 years. In 2019, there were more than 400 million UTIs cases worldwide, which represented an increase of 60.40% from the figure in 1990 (Zhu et al., 2021). UTIs are often treated with antibiotics (Foxman, 2014), but with the spread of antibiotic resistance among Gram-negative bacteria (Echols et al., 1999), bacteria are likely to develop resistance to the antibiotics commonly used for UTIs. UTIs are classified into stranguria in traditional Chinese medicine, and Chinese herbal medicines have the advantages of good curative effects, low toxicity, and low recurrence rates in treating UTIs (Li et al., 2019).

Sanjin tablets (SJT), a Chinese patent drug, have been used to treat UTIs clinically for nearly 40 years. They are produced by a water decoction of five herbs, i.e., Rosae Laevigatae Radix (RLR), Smilacis Chinae Rhizoma (SCR), Melastoma Radix et Rhizoma (MRR), Lygodii Herba (LH), and Centellae Herba (CH). Their effects of clearing up heat and toxicity, removing dampness, treating stranguria, and tonifying the kidney are often used for the treatment of hot stranguria, reddish urine, and the painful, urgent, and frequent urination caused by damp heat in the lower jiao, as well as acute or chronic pyelonephritis, cystitis, and UTIs with these symptoms and signs (State Pharmacopoeia Commission, 2020). A meta-analysis showed a good effect of SJT on UTIs (Lyu et al., 2019; Lyu et al., 2020), but the underlying mechanism of action remains unclear. SJT were first listed in the 2000 edition of the *Chinese Pharmacopoeia*. Since then, the quality standard of this drug has been continuously improved. The current standard of content measurement is based on the content of madecassoside in each tablet, but this single index compound is not sufficient to characterize the overall quality of this drug. The efficacy of this Chinese patent drug is well proven, but there has been little basic research on it as of yet. Only 32 constituents have been identified in SJT (Meng et al., 2015; Zou, 2019; State Pharmacopoeia Commission, 2020; Yang, 2021; Zou et al., 2021), and the chemical constituents and effective substances of this drug remain unknown. A study of the chemical constituents and effective substances of SJT may help improve their quality and promote further research and development.

A Chinese patent drug is usually composed of many kinds of Chinese herbs, and each herb contains different chemical constituents, which act on different targets and exert their therapeutic effects via different pathways (Yuan et al., 2017). Identification of the chemical constituents and action targets is the key to scientific research on Chinese herbal medicines.

HPLC-ESI-IT-TOF-MS^n^ is a fast analytical technique with high sensitivity, high resolution, and high selectivity, which allows for a comprehensive analysis and identification of multicomponent systems and was adopted in many studies on the composition of Chinese herbs (Gao et al., 2016; Wang et al., 2020). Network pharmacology combines network biology and pharmacology and, in the context of interlinked biological networks and pathways, can be used to study the interactions among drugs, targets, and diseases and thus is in agreement with the multicomponent–multitarget–multipathway characteristics of Chinese herbal medicines; it has been widely used to identify active compounds and explore potential therapeutic mechanisms of Chinese herbal medicines (Zhai et al., 2020; Zhou et al., 2021).

In this study, HPLC-ESI-IT-TOF-MS^n^ was used to identify the chemical constituents of SJT, and network pharmacology was used to predict the targets of action of these constituents to elucidate the effective compounds and functional mechanism of SJT in the treatment of UTIs. AutoDock Vina was used to evaluate the affinities between key effective substances and core targets.

## 2 Materials and methods

### 2.1 Instruments and materials

The instruments used included a Shimadzu LC-ESI-IT-TOF-MS spectrometer (Shimadzu, Kyoto, Japan); an HPLC system (Shimadzu, Kyoto, Japan): DGU-20A3 on-line degasser, two LC-20AD pumps, SIL-20AC autosampler, CTO-20 AC column oven, SPD-M20A diode array detector, CBM-20 A controller; a Gemini-NX C_18_ LC column (4.6 × 250 mm, 5 µm particle size) (Phenomenex, Torrance, United States). KQ-500DE Ultrasonic washer (Kunshan Ultrasonic Instrument Co., Ltd., Kunshan, China); Milli-Q Integral 3 ultrapure water machine (Milli-Q, Billerica, MA, United States); and a Sartorius 1/100,000 electronic balance (Sartorius, Gottingen, Germany). The chemical compounds used included methanol (HPLC grade, Beijing Tongguang Fine Chemical Company, lot 20210302), acetonitrile (HPLC grade, Thermo, lot 204681), and formic acid (HPLC grade, Thermo, lot 212271).

SJT were purchased from Guilin Sanjin Pharmaceutical Co., Ltd. (Lot: 210417). The reference compounds were isorhamnetin-3-*O*-β-D-glucoside (Lot: PS011411; 99.55%), epicatechin (Lot: PS010585), asiaticoside B (Lot: PS010252) (purity ≥99.0% for all), kaempferol-7-*O*-β-D-glucoside (Lot: PS012598), 1,3-dicaffeoylquinic (Lot: PS012091) (purity ≥98.5% for all), rutin (Lot: PS012206), catechin (Lot: PS020094), malic acid (Lot: PS010539), kaempferol (Lot: PS011599), mikwelianin (Lot: PS011018), afzelin (Lot: PS011206), kaemferol-3-*O*-β-D-glucoside (Lot: PS011379), quercetin 3-*O*-a-L-rhamnopyranoside (Lot: PS010791), engeletin (Lot: PS012273), isoquercetin (Lot: PS001042), procyanidin B3 (Lot: PS012656), kaempferitrin (Lot: PS020385), vicenin-2 (Lot: PS012054), nicotflorin (Lot: PS011341), isochlorogenic acid B (Lot: PS001054), isochlorogenic acid A (Lot: PS001052), 1,5-dicaffeoylquinic Acid (Lot: PS011115), neoastilbin (Lot: PS010667), astilbin (Lot: PS000649), neoisoastilbin (Lot: PS010708), isoastilbin (Lot: PS011862), caffeic acid (Lot: PS010522), gallic acid (Lot: PS000688), phlorizin (Lot: PS020349), euscaphic acid (Lot: PS012455), madecassoside (Lot: PS000752), madecassic acid (Lot: PS020268) purity ≥98.0% for all, aromadendrin (Lot: PS012280), tormentic acid (Lot: PS011285) purity ≥95.0% for all, and 19α-hydroxyasiatic acid (Lot: PS011490; ≥90.0%). The abovementioned reference compounds were purchased from Chengdu Push Biotechnology Co., Ltd. (Chengdu, China). Quercetin (Lot: JC07P829; purity ≥97%) was purchased from Zancheng Technology Co., Ltd. (Tianjin, China). Neochlorogenic acid (Lot: PCS-210509), chlorogenic acid (Lot: PCS-220413), cryptochlorogenic acid (Lot: PCS-210712), 5-*O*-caffeoylshikimic acid (Lot: PCS-220314), and 1,4-dicaffeoylquinic acid (Lot: PCS-220314) (purity ≥98.0% for all) were purchased from Chengdu HerbSubstance Biotechnology Co., Ltd., China. Ellagic acid (Lot: JC07P829; 98% of purity) was purchased from Shanghai Hongbai Technology Co., Ltd. (Shanghai, China); isochlorogenic acid C (Lot: MUST-21081010; purity ≥98.0%) was purchased from Chengdu Must Biotechnology Co., Ltd. (Chengdu, China), and asiaticoside (Lot: 110892–202006; purity ≥93.8%) was purchased from the China National Institutes for Food and Drug Control.

### 2.2 Experimental methods

#### 2.2.1 Preparation of test solutions

##### 2.2.1.1 SJT sample solution

Twenty SJT were removed from their coating, accurately weighed, and ground; 1.5 g of the powder was accurately weighed, added to 50 mL methanol, and weighed once again. The mixture was ultrasonicated for 45 min, cooled to room temperature, and weighed. The lost weight was made up with methanol. The solution was shaken well and filtered; 25 mL of the filtrate was accurately measured, followed by recovery of the solvent to dryness. The sample was resolved with methanol and transferred to a 5 mL volumetric flask, followed by the addition of methanol to volume. The solution was shaken well, and 1 mL of the sample was transferred into an EP tube, dried by blowing nitrogen, dissolved with 100 µL of methanol, and filtered through a 0.22 μm microporous membrane. The resulting filtrate was used for the subsequent analysis.

##### 2.2.1.2 Preparation of reference compound solutions

To prepare the pooled reference compound solutions, 44 reference compounds were divided into seven groups so that isomers were not in the same group. Group 1: catechin, neochlorogenic acid, malic acid, kaemferol-3-*O*-β-D-glucoside, isorhamnet-in-3-*O*-β-D-glucoside, isochlorogenic acid A, neoisoastilbin, and tormentic acid; group 2: kaempferol-7-*O*-β-D-glucoside, isochlorogenic acid B, isoastilbin, and asiaticoside B; group 3: kaempferol, procyanidin B3, vicenin-2, isochlorogenic acid C, neoastilbin, and madecassoside; group 4: mikwelianin, kaempferitrin, nicotflorin, 1,5-dicaffeoylquinic acid, astilbin, aromadendrin, and madecassic acid; group 5: chlorogenic acid, afzelin, 1,3-dicaffeoylquinic, caffeic acid, phlorizin, euscaphic acid and 19α-hydroxyasiatic acid; group 6: rutin, engeletin, 5-*O*-caffeoylshikimic acid, and asiaticoside; group 7: quercetin, epicatechin, cryptochlorogenic acid, quercetin 3-*O*-a-L-rhamnopyranoside, isoquercetin, ellagic acid, 1,4-dicaffeoylquinic acid, and gallic acid. Each reference compound (2 mg) was dissolved in 200 µL of methanol, 150 µL of each reference compound was added to the corresponding group, and the final volume of each group was adjusted to 1.5 mL with methanol. The solution was filtered through a 0.22 μm microporous membrane, and the filtrate was used for subsequent analysis.

#### 2.2.2 LC-MS conditions

A Gemini-NX C_18_ LC column (4.6 mm × 250 mm, 5 µm) was used; mobile phase: 0.1% formic acid–water solution (A)–acetonitrile (B) with gradient elution as follows: 0%–3% B (0–7.5 min), 3%–13% B (7.5–127.5 min), 13%–17% B (127.5–130.5 min), isocratic 17% B (130.5–145.5 min), 17%–20% B (145.5–195.0 min), 20%–25% B (195.0–202.5 min), 25%–32% B (202.5–265.5 min), 32%–60% B (265.5–285.0 min), 60%–100% B (285.0–315.0 min), isocratic 100% B (315.0–330.0 min); PDA recording wavelength: 195–400 nm; flow rate: 1 mL/min; injection volume: 10 μL; column temperature: 30°C.

MS conditions: 0.2000 mL/min split from HPLC; ESI ion source; detection mode: positive ion (PI) and negative ion (NI); detection range: MS, *m*/*z* 100–2000; MS^2^ and MS^3^, *m*/*z* 50–2000; ion accumulation time 20–30 ms; nebulizing gas (N_2_) flow rate: 1.5 L/min; curved desolvation line and heating block temperature at 250 °C; detection voltage: 1.73 kV; drying gas (N_2_) pressure: 100 kPa.

#### 2.2.3 Methods of compound identification

The chemical constituents of SJT were analyzed by the HPLC-ESI-IT-TOF-MS^n^ technique, and the collected data were processed by the LCMSSolution workstation. According to the quasi-molecular ion peaks measured in the negative ion mode, the accurate molecular mass was determined, and the molecular composition was calculated using the MS analysis software. Based on a comparison of the theoretical and measured values and consultation of the SJT-DB and relevant literature, the fragmen-tation pathways of these constituents were analyzed, and SciFinder was consulted to speculate the structure of the compounds. The structure of some compounds was further confirmed by comparison with their reference compounds.

#### 2.2.4 Construction of the “herb–compound–target” network

To obtain the potential targets of SJT-MS and SJT-DB, the compounds were imported into the SuperPred database (https://prediction.charite.de/subpages/target_prediction.php/, accessed on 1 January 2023) (Nickel et al., 2014) and the SwissTargetPrediction database (https://SwissTargetPrediction.ch/, accessed on 1 January 2023) (Daina et al., 2019).

The targets associated with UTIs were obtained from the DisGeNET database (https://www.disgenet.org/search, accessed on 1 January 2023) (Piñero et al., 2017) and the GeneCards database (https://www.genecards.org/, accessed on 1 January 2023) (Stelzer et al., 2016) using the keywords “Urinary tract infection” and “*Homo sapiens*” as a filter. Moreover, the targets in GeneCards were filtered according to more than twice the median.

The intersection of the SJT-MS and UTI related targets (common targets) was selected to construct a “herb–compound–target” network using Cytoscape 3.9.1.

#### 2.2.5 Construction of the protein–protein interaction (PPI) network

The common targets obtained in Section 2.2.4 were imported into the STRING database (https:///STRING db. org/, accessed on 4 January 2023), and the protein type was set as *H. sapiens*. In order to ensure the reliability of the data, the minimum interaction score was set to 0.7, and a PPI network diagram was obtained and exported in SVG format. The exported results were imported into Cytoscape software, and the CytoNCA plug-in was used to calculate the degree value of the network for core target screening.

#### 2.2.6 Gene ontology (GO) and KEGG pathway enrichment analyses

The core targets obtained from Section 2.2.5 were imported into the Metascape database (http://metascape.org/gp/index.html#/main/step1, accessed on 1 January 2023) (Zhou et al., 2019) to elucidate the functions of the targets and their functions in signaling transduction.

#### 2.2.7 Construction of the “potential effective compound–core target–signaling pathway” network

According to the compound–target relationship in Section 2.2.4, compounds corresponding to the core target in Section 2.2.5 were perceived as potential effective compounds of SJT and were integrated with the relationship between the core target and KEGG signal pathway in Section 2.2.6. The “potential effective compound–core target–signaling pathway” network was constructed using Cytoscape 3.9.1 software.

#### 2.2.8 Molecular docking between 12 key effective substances and 10 core targets

The potential effective compounds that have been reported to possess antimicrobial and anti-inflammatory activities in the literature are regarded as key effective substances, and their structures in SDF file format were downloaded from the Pub-Chem database (http://pubchem.ncbi.nlm.nih.gov, accessed on 22 January 2023). The top nine potential targets in the PPI network and PIT1 (Pang et al., 2022) were selected as receptor proteins, and the crystal PDB structure file was searched in the Protein Data Bank (PDB) database (http://www.rcsb.org/, accessed on 10 January 2023). The protein names and PDB IDs were as follows: HSP90AA1 (3O0I), SRC (6ATE), STAT3 (6NJS), AKT1 (7NH5), EGFR (5UG9), PIK3R1 (5GJI), VEGFA (4KZN), PTPN11 (3ZM1), MAPK1 (2Y9Q), and PIT1 (5WC9). AutoDock Vina 1.1.2 software was used for molecular docking; the binding strength of core targets and key effective substances was evaluated based on the docking score, and PyMOL software was used for visual analysis.

## 3 Results

### 3.1 Establishment of the compound database of SJT (SJT-DB)

The SJT-DB was established using ChemOffice by integrating the structure, molecular formula, molecular weight, Chinese name, English name, MS fragments, CAS number, the literature, and other data on each of the reported compounds of the five Chinese herbs of which SJT are composed (Liang et al., 2013). Searching the CNKI, SciFinder, and Pubchem databases resulted in a total of 32 SJT constituents, 244 CH constituents, 206 SCR constituents, 61 LH constituents, 90 RLR constituents, and 38 MRR constituents. After duplicates were removed, there were a total of 618 compounds in SJT-DB.

### 3.2 Characterization of the constituents of SJT by HPLC-ESI-IT-TOF-MS^n^

**FIGURE 1:**
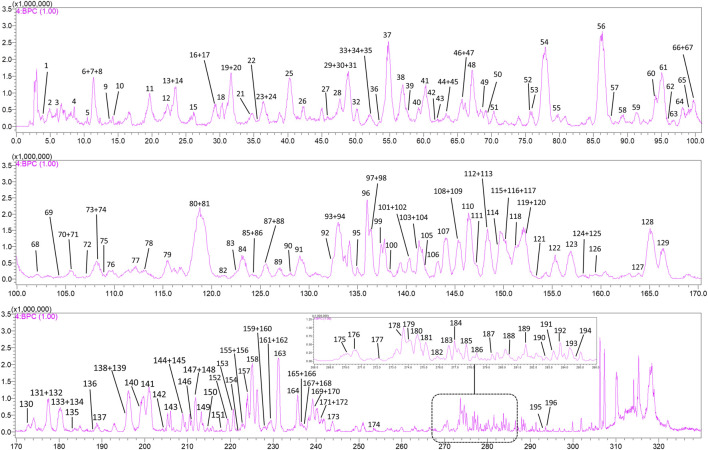
Negative ion base peak chromatogram (BPC) of SJT with 196 labeled compounds. The top 10 compounds in peak area are: C56, arthromerin B or helicioside A; C81, fisetinidol-(4a → 8)-catechin or isomer; C61*, 1,3-dicaffeoylquinic acid; C54, eriodictyol 3′-*O*-β-D-glucoside; C119*, 1,5-dicaffeoylquinic acid; C128*, isochlorogenic acid C; C93*, neoastilbin; C30, 6-*O*-caffeoyl-β-D-glucopyranose or isomer; C25, 6-*O*-caffeoyl-β-D-glucopyranose or isomer; C138, (2α,3β,4β,19α)-23-(glycero-manno-Heptonoyloxy)-2,3,19-trihydroxyolean-12-en-28-oic acid or isomer.

**FIGURE 2:**
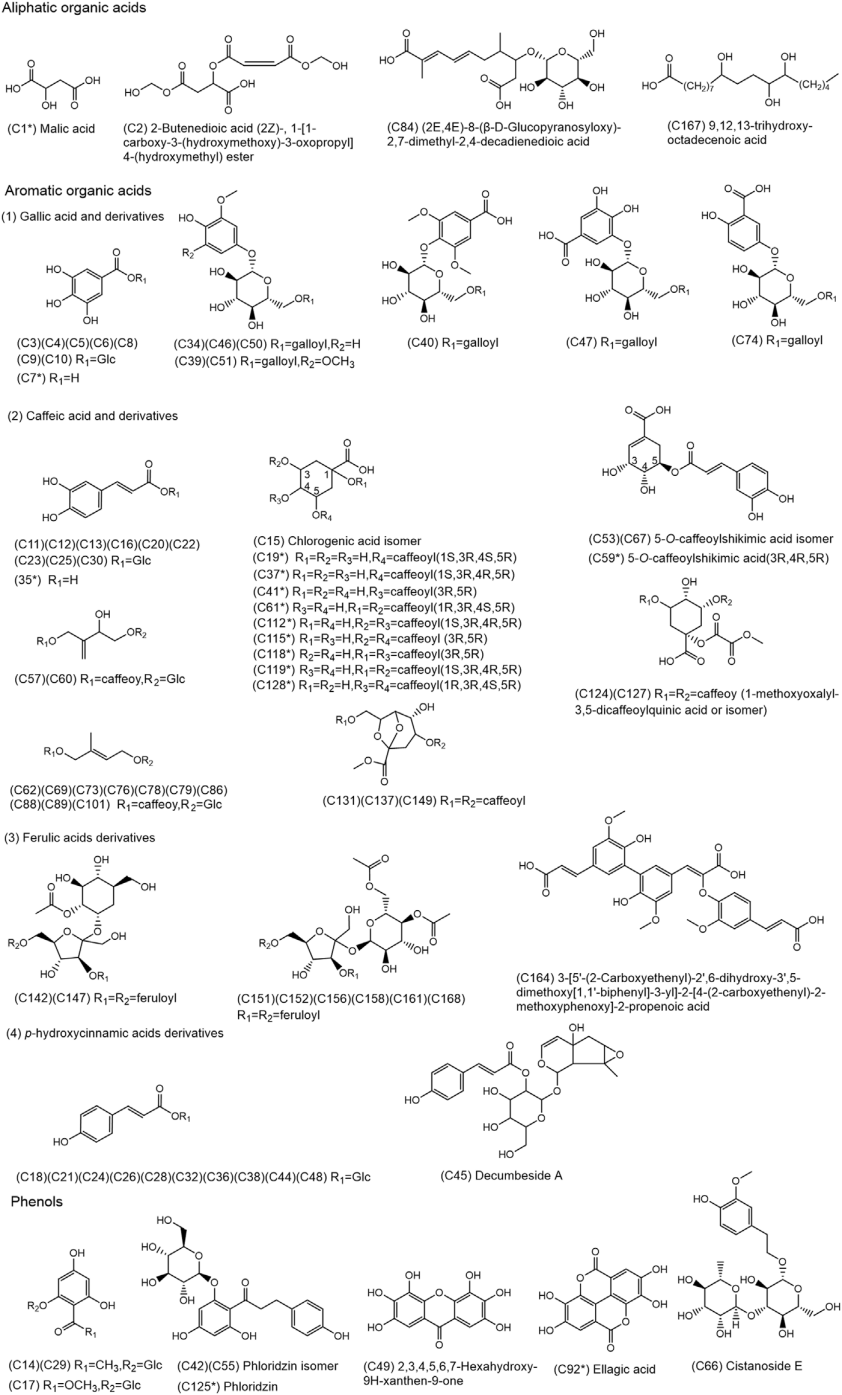
Chemical structures of the 80 organic acids and 9 phenols identified in SJT. (* Compounds confirmed by comparison with reference compounds).

**FIGURE 3:**
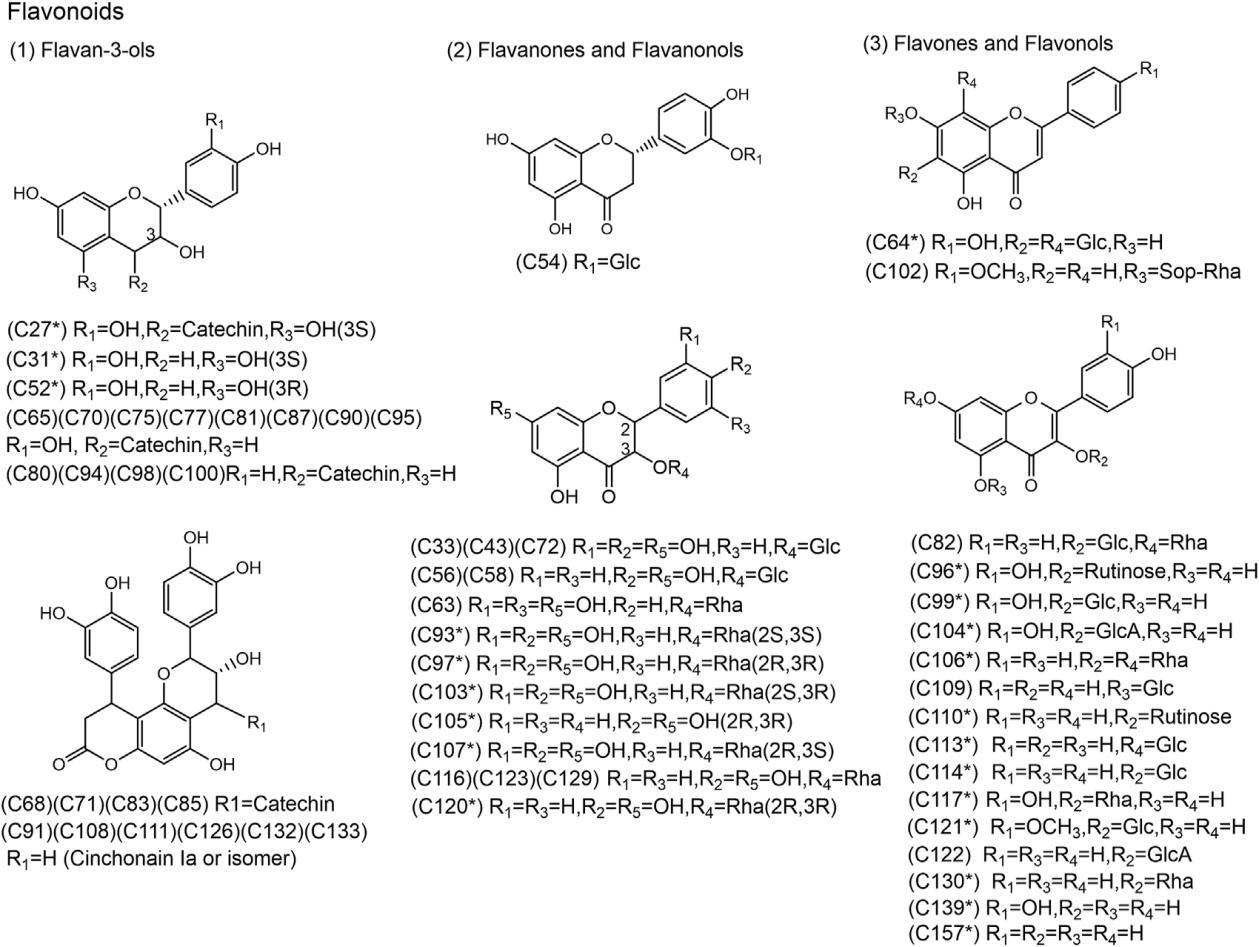
Chemical structures of the 58 flavonoids identified in SJT. (* Compounds confirmed by comparison with reference compounds).

**FIGURE 4:**
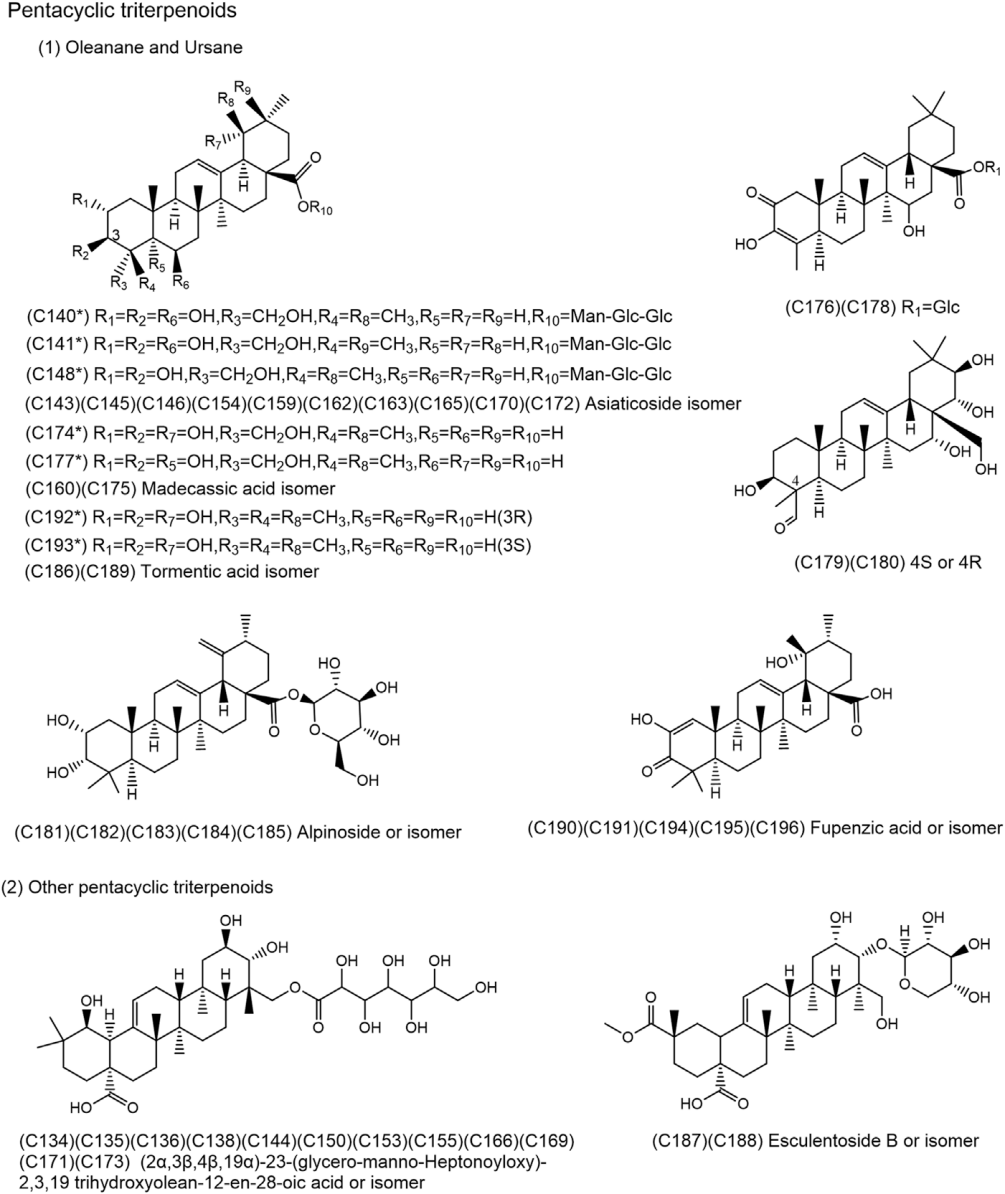
Chemical structures of the 49 pentacyclic triterpenoids identified in SJT. (*Compounds confirmed by comparison with reference compounds).

A total of 196 compounds were identified in the methanol extract of SJT, of which 13 were potential new compounds. Among the 183 known compounds, 169 were new chemical constituents of SJT, and 93 were not reported in their five constituent herbs. Figure 1 is the negative ion base peak chromatogram (BPC) of the methanol extract of SJT, and the specific information of the 196 compounds is detailed in Supplementary Table S1 and Figures 2–4.

### 3.3 MS fragmentation and structural analysis of the chemical constituents of SJT

#### 3.3.1 Identification of aliphatic organic acids

Compound 1 (C1) showed [M−H]^−^ at *m*/*z* 133.0153 in the MS^1^ spectra. Its molecular formula was predicted to be C_4_H_6_O_5_, and the fragment ion [M−H−H_2_O]^−^ at *m*/*z* 115.0183 was observed in the MS^2^ spectra, implying that the compound contains a hydroxyl group. The compound was confirmed to be malic acid by consulting the literature (Ju et al., 2021) and comparison with the reference substance. The molecular formula of C2 is C_10_H_12_O_10_, and the fragment ions [M−H−C_5_H_6_O_5_]^−^ at *m*/*z* 145.0045 and [M−H−C_5_H_6_O_5_−H_2_O]^−^ at *m*/*z* 127.0144 were present in the MS^2^ spectra. The compound was presumed to be 2-butenedioic acid (2Z)-, 1-[1-carboxy-3-(hydroxymethoxy)-3-oxopropyl] 4-(hydroxymethyl) ester by consulting the SciFinder database; the neutral molecule C_5_H_6_O_5_ was lost because the ester bond is easily broken, and the hydroxyl group was lost by dehydration. In the MS^2^ spectra of C84, the fragment ions [M−H−C_6_H_12_O_6_]^−^ at *m*/*z* 223.1017, and [M−H−C_6_H_12_O_6_−CO_2_]^−^ at *m*/*z* 179.1038 were present. Hence, C84 was presumed to contain glucosyl and carboxyl, and it was presumed to be (2E, 4E)-8-(β-D-glucopyranosyloxy)-2,7-dimethyl-2,4-decadienedioic acid or its isomer by consulting the SciFinder database. In the MS^2^ spectra of C167, the fragment ions [M−H−H_2_O]^−^ at *m*/*z* 313.2420, [M−H−2H_2_O]^−^ at *m*/*z* 295.2367, and [M−H−C_9_H_20_O_2_]^−^ at *m*/*z* 171.1027 were present. Hence, C167 was presumed to be 9,12,13-trihydroxy-octadecenoic acid, a compound of the SJT-DB that contains three hydroxyl groups, which are possibly lost through dehydration. C_9_H_20_O_2_ may be lost by the *β*-bond fragmentation of hydroxyl groups.

#### 3.3.2 Identification of aromatic organic acids

##### 3.3.2.1 Identification of gallic acid and its derivatives

Most of these compounds in SJT consist of gallic acid (C_7_H_6_O_5_, M = 170.02) and glucose (C_6_H_12_O_6_, M = 180.06), which form galloylglucose, with phenolic structures occasionally attached to the saccharide structure. Mass spectrometry frequently reveals the loss of phenolic structural units, followed by the loss of glucose, to result in the molecular ion ([M−H]^−^) of gallic acid at *m*/*z* 169.01.

C3–C6 and C8–C10 are isomers of galloylglucose compounds. The position where galloyl links to glucose differs among the seven compounds. [M−H]^−^ at *m*/*z* 331.06 is present in their MS^1^ spectra, and the ion of gallic acid at *m*/*z* 169.01 is present in their MS^2^ spectra. In addition, the fragment ions at *m*/*z* 271.05 and *m*/*z* 211.02 resulting from saccharide ring fragmentation can be seen.

The fragment ion [M−H−CO_2_]^−^ at *m*/*z* 125.0239 was observed in the MS^2^ spectra of C7; hence, C7 was presumed to contain carboxyl groups and was confirmed to be gallic acid by comparison with the reference substance.

C34, C39, C46, C50, and C51 showed the galloylhexosyl ion at *m*/*z* 313.05 and the gallic acid ion at *m*/*z* 169.01 in their MS^2^ spectra, and they were presumed to be galloylphenol glycosides. There was an extra −OCH_2_(30 Da) in C39 and C51 when compared to C34, C46, and C50; hence, it was presumed that there was one additional methoxy group on the phenolic group linked to the C39 and C51 galloyl glycosides. They were preliminarily identified as (3,5-dimethoxy-4-hydroxyphenyl)-1-*O*-β-D-(6-*O*-galloyl) glucopyranoside and (4,5-dimethoxy-3-hydroxyphenyl)-1-*O*-β-D-(6-*O*-galloyl) glucopyranoside by consulting the SciFinder database.

C40, C47, and C74 showed fragment ion [M−H−CO_2_]^−^ at *m*/*z* 467.1142, m/*z* 439.0857, and *m*/*z* 423.0898, respectively, in their MS^2^ spectra; hence, they were presumed to contain carboxyl groups. In addition, the galloylhexosyl ion at *m*/*z* 313.05 and gallic acid ion at *m*/*z* 169.01 were observed. Based on consultation of the SciFinder database, the three compounds were presumed to be 3,5-dimethoxy-4-[[6-*O*-(3,4,5-trihydroxybenzoyl)-β-D-glucopyranosyl]oxy]benzoic acid, gallic acid 3-*O*-(6′-*O*-galloyl)-β-D-glucopyranoside, and 5-*O*-(6′-*O*-galloyl-β-D-glucopyranosyl)gentisic acid or its isomer.

##### 3.3.2.2 Identification of caffeic acid and its derivatives

The main structural feature of these compounds in SJT is the binding of one or two molecules of caffeic acid (C_9_H_8_O_4_, M = 180.04) to glucose (M = 180.06), quinic acid (C_7_H_12_O_6_, M = 192.06), or shikimic acid (C_7_H_10_O_5_, M = 174.05).

C11–13, C16, C20, C22, C23, C25, and C30 are a group of isomers (C_15_H_18_O_9_). [M−H]^−^ at *m*/*z* 341.09 is present in their MS^1^ spectra, and the caffeic acid ion (C_9_H_7_O_4_, [M−H−Glc]^−^) at *m*/*z* 179.03 is present in their MS^2^ spectra. In addition, fragments at *m*/*z* 281.06 and *m*/*z* 251.05 formed by saccharide ring fragmentation could be seen, which agrees with the structure of caffeoylglucose compounds of the SJT-DB. It was presumed that the structure of this group of compounds differs in terms of the linking position of caffeoyl and glucose.

C35 showed [M−H]^−^ at *m*/*z* 179.0338 in the MS^1^ spectra and the characteristic fragment ion [M−H−CO_2_]^−^ of caffeic acid at *m*/*z* 135.0499 in the MS^2^ spectra. C35 was confirmed to be caffeic acid by comparison with the reference substance.

C15, C19, C37, and C41 are a group of isomers. The ion [M−H]^−^ at *m*/*z* 353.09 can be seen in their MS^1^ spectra, and caffeic acid ion at *m*/*z* 179.04, quinine acid ion at *m*/*z* 191.06, and the characteristic fragment ions of caffeic acid at *m*/*z* 135.04 and 161.02 are present in their MS^2^ spectra. Based on the literature (Demarque et al., 2016; De Rosso et al., 2018), this group of compounds was presumed to be chlorogenic acid and its isomers. By comparison with the reference substances, C19, C37, and C41 were confirmed to be neochlorogenic acid, chlorogenic acid, and cryptochlorogenic acid, respectively, and C15 was presumed to be their isomer.

C61, C112, C115, C118, C119, and C128 are isomers of isochlorogenic acids; by comparison with the reference substances, they were identified to be 1,3-dicaffeoylquinic acid, isochlorogenic acid B, isochlorogenic acid A, 1,4-dicaffeoylquinic acid, 1,5-dicaffeoylquinic acid, and isochlorogenic acid C, respectively.

C53, C59, and C67 are a group of isomers, and the [M−H]^−^ at *m*/*z* 335.07 can be seen in their MS^1^ spectra, and caffeic acid ion at *m*/*z* 179.0388 and caffeic acid fragment ions at *m*/*z* 135.04 and *m*/*z* 161.02 can be seen in their MS^2^ spectra. Hence, they were presumed to be 5-*O*-caffeoylshikimic acid and its isomers of the SJT-DB. C59 was identified as 5-*O*-caffeoylshikimic acid by comparison with the reference substance.

C62, C69, C73, C76, C78, C79, C86, C88, C89, and C101 are 10 isomers (C_20_H_26_O_10_), and the characteristic fragments of caffeic acid were observed in their MS^2^ spectra. Only eight compounds that agree with these characteristic fragments were found in the SciFinder database: rotundarpenoside B and its isomers; the other two compounds were presumed to be new compounds.

C124 and C127 are a pair of isomers, and the [M−H]^−^ at *m*/*z* 601.12 can be seen in the MS^1^ spectra. The structure of 1-methoxyoxalyl-3,5-dicaffeoylquinic acid was found to be consistent with the fragments found in the MS^2^ spectra by consulting the SciFinder database. There is a fragmentation process of the loss of neutral molecules CO_2_ and H_2_O and the loss of substituents via the breakage of ester and ether bonds.

C131, C137, and C149 are a group of isomers. The structure of erigoster A was found by consulting the SciFinder database, which showed agreement with the fragment seen in their MS^2^ spectra. The structure was composed of a 6,8-dioxabicyclo octane ring linked by two caffeic acid molecules. The neutral loss of H_2_O and ester bond cleavage were observed in their MS^2^ spectra. Therefore, they are erigoster A or its isomers.

##### 3.3.2.3 Identification of ferulic acid derivatives

SJT mainly comprise ferulate esters and ferulic acid (M = 194.06) oligomers. C142 and C147 showed the ion [M−H]^−^ at *m*/*z* 735.21, and their molecular formula was predicted to be C_34_H_40_O_18_. Based on the fragment ions in the MS^2^ spectra, the two compounds were presumed to be smilaside B and its isomer of the SJT-DB. This structure is formed by two ferulic acid molecules linked by a disaccharide. The fragmentation pathway of smilaside B mainly involves saccharide ring cleavage, branch chain cleavage, ether bond cleavage, and the loss of H_2_O.

C151, C152, C156, C158, C161, and C168 are a group of isomers. Based on the fragment ions in the MS^2^ spectra, they were presumed to be smilaside A and its isomer of the SJT-DB. Smilaside A has an extra C_2_H_2_O molecule compared with smilaside B. The fragmentation pathway mainly involves ester bond cleavage and dehydration.

C164 showed [M−H]^−^ at *m*/*z* 577.14, and its molecular formula was presumed to be C_30_H_26_O_12_. The ferulic acid ion at *m*/*z* 193.0530 and ferulic acid dimer ion at *m*/*z* 385.0846 can be seen in the MS^2^ spectra. Therefore, C164 was presumed to be a ferulic acid trimer. It was preliminarily identified as 3-[5′-(2-Carboxyethenyl)-2′,6-dihydroxy-3′,5-dimethoxy [1,1′-biphenyl]-3-yl]-2-[4-(2-carboxyethenyl)-2-methoxyphenoxy]-2-propenoic acid by consulting the SciFinder database.

##### 3.3.2.4 Identification of *p*-hydroxycinnamic acid derivatives

The main feature of these compounds in SJT is the formation of hydroxycinnamyl glucose from hydroxycinnamic acid (C_9_H_8_O_3_, M = 164.05) and glucose (M = 180.06).

C18, C21, C24, C26, C28, C32, C36, C38, C44, and C48 are a group of isomers, and the ion [M−H]^−^ at *m*/*z* 325.09 can be seen in their MS^1^ spectra. The characteristic fragment ions [M−H−H_2_O]^−^ at *m*/*z* 307.08, [M−H−CH_2_O]^−^ at *m*/*z* 295.08, [M−H−2CH_2_O]^−^ at *m*/*z* 265.07 and [M−H−Glc]^−^ at *m*/*z* 163.04 formed by saccharide ring fragmentation and glycosidic bond fragmentation can also be seen in their MS^2^ spectra. They were presumed to be hydroxycinnamyl glucose or its isomers by consulting the SciFinder database.

C45 showed the *p*-hydroxy cinnamic acid ion at *m*/*z* 163.04 and *p*-hydroxy cinnamyl glucosyl ion at *m*/*z* 325.09 in the MS^2^ spectra, and it was preliminarily identified as decumbeside A by consulting the SciFinder database.

#### 3.3.3. Identification of other phenols

C14 and C29 showed the quasi-molecular ion [M−H]^−^ at *m*/*z* 329.09 in their MS^1^ spectra, and they were presumed to be myrciaphenone A and its isomers of the SJT-DB. In the MS^2^ spectra, the 2,4,6-trihydroxyacetophenone ion at *m*/*z* 167.03 formed by the glycosidic bond cleavage of myrciaphenone A was observed.

C17 showed the 2,4,6-trihydroxybenzoic acid ion at *m*/*z* 169.0145 in the MS^2^ spectra, and it was presumed to be methyl 2-(β-D-glucopyranosyloxy)-4,6-dihydroxybenzoate of the SJT-DB.

C42, C55, and C125 are a group of isomers presumed to be phloridzin and its isomers of the SJT-DB based on the fragments observed in the MS^2^ spectra. C125 was identified as phloridzin by consulting the literature (Fan et al., 2020) and in comparison with the reference substance.

C49 showed fragment ions [M−H−CO_2_]^−^ at *m*/*z* 247.0250, [M−H−CO_2_−CO]^−^ at *m*/*z* 219.0252, and [M−H−CO_2_−2CO]^−^ at *m*/*z* 191.0353 in the MS^2^ spectra, and it was presumed to be 2,3,4,5,6,7-hexahydroxy-9H-xanthen-9-one by consulting the SciFinder database.

C66 showed [M−H]^−^ at *m*/*z* in the MS^1^ spectra, the rhamnose ion at *m*/*z* 163.06, and fragment ions at *m*/*z* 265.09 and 235.08, formed by saccharide ring cleavage in the MS^2^ spectra. It was presumed to be cistanoside E or its isomer by consulting the SciFinder database.

C92 showed [M−H]^−^ at *m*/*z* 300.9968 in the MS^1^ spectra; its molecular formula was predicted as C_14_H_6_O_8_, and fragments resulting from continuous loss of CO and CO_2_ are present in the MS^2^ spectra. C92 was confirmed as ellagic acid by consulting the literature (Okba et al., 2021) and in comparison with the reference substance.

#### 3.3.4 Identification of flavonoids

##### 3.3.4.1 Identification of catechin and its derivatives

Procyanidins are formed by binding various numbers of catechin (C_15_H_14_O_6_, M = 290.08) or epicatechin (C_15_H_14_O_6_, M = 290.08). C27 showed [M−H]^−^ at *m*/*z* 577.1350 in the MS^1^ spectra; its molecular formula was predicted as C_30_H_26_O_12_ and it was confirmed to be procyanidin B3 by consulting the literature (Huang et al., 2021) and in comparison with the reference substance.

C31 and C52 are a pair of isomers identified as catechin and epicatechin, respectively, by consulting the literature (Grace et al., 2014) and in comparison with the reference substances. By analyzing the MS^2^ spectra of the reference substances, it was found that the MS fragmentation process of catechin and epicatechin mainly involved the loss of CO_2_ from ring A and continuous losses of C_2_H_2_O from ring B.

C65, C70, C75, C77, C81, C87, C90, and C95 are a group of isomers (C_30_H_26_O_11_), and the characteristic fragment ions of catechins, including [M−H−C_6_H_6_O_2_]^−^ at *m*/*z* 451.10 (loss of ring B), the fragment ion at *m*/*z* 409.09 (RDA fragmentation of ring C after loss of ring B), and catechin at *m*/*z* 289.07, are present in their MS^2^ spectra. They were presumed to be fisetinidol-(4a → 8)-catechin, fisetinidol-(4β → 8)-catechin, ent-fisetinidol (4β → 6)-catechin, and their isomers of the SJT-DB.

C68, C71, C83, and C85 are a group of isomers with catechin fragmentation behaviors such as the loss of ring B, loss of C_2_H_2_O from ring C, ester bond cleavage, and loss of H_2_O, and they were presumed to be cinchonain IIa, cinchonain IIb, and their isomers of the SJT-DB by consulting the literature (Brahmi-Chendouh et al., 2019).

##### 3.3.4.2 Identification of dihydroflavones and dihydroflavonols

The dihydroflavonols in SJT are mostly in the form of free dihydroflavonol or *O*-monoglycosides. The glycosylation site is usually at C-3 hydroxyl in these compounds, and the aglycon ion can be formed by glycosidic bond cleavage. The aglycone ion is prone to typical Retro Diels–Alder (RDA) fragmentation (Wu et al., 2004).

C54, C56, C58, and C63 showed [M−H]^−^ at *m*/*z* 449.11 in their MS^1^ spectra, and their molecular formula was predicted as C_21_H_22_O_11_. [M−H−Glc]^−^ at *m*/*z* 287.05 was observed in their MS^2^ spectra, and RDA fragmentation or the loss of carbonyl occurred in ring C. They were presumed to be eriodictyol 3′-*O*-β-D-glucoside, arthromerin B, helicioside A, and 3,5,7,3′,5′-pentahydroxy-2R,3R-flavanonol 3-*O*-α-L-rhamnopyranoside of the SJT-DB.

C33, C43, and C72 were presumed to be (2R, 3R)-taxifolin 3*-O*-β-D-glucopyranoside and its isomers of the SJT-DB. The MS^2^ spectra showed the fragment ion [M−H−Glc]^−^ at *m*/*z* 303.05 resulting from the loss of glycosyl, the fragment ion at *m*/*z* 285.04 resulting from the subsequent loss of 3-OH from ring C, the fragment ion at *m*/*z* 241.05 resulting from the subsequent loss of CO_2_ from ring B, or the fragment ion at *m*/*z* 175.00 resulting from the loss of ring B.

C93, C97, C103, and C107 are a group of isomers, which were confirmed to be neoastilbin, astilbin, neoisoastilbin, and isoastilbin, respectively, by comparison with the reference substances. By consulting the literature (Zhang et al., 2013) and through analysis of the MS^2^ spectra of the reference substances, it was presumed that the *o*-diphenol hydroxyl in ring B of dihydroflavonol is prone to dehydration or is lost as a molecule of CO_2_, and the loss of a molecule of water or carbonyl or RDA fragmentation occurs in ring C.

C105 showed [M−H]^−^ at *m*/*z* 287.0549 in the MS^1^ spectra, and its molecular formula was predicted as C_15_H_12_O_6_. [M−H]^−^ at *m*/*z* 287.06, with three main fragmentation pathways. The first is that *m*/*z* 287.06 loses a molecule of H_2_O to form a fragment ion at *m*/*z* 269.05. The second is that ring C loses a molecule of CO to form a fragment ion at *m*/*z* 259.06. The third is that ring C loses one molecule of CO_2_ to form a fragment ion at *m*/*z* 243.07, which loses one molecule of C_2_H_2_O from ring B to form a fragment ion at *m*/*z* 201.06, which loses one molecule of C_2_H_4_O_3_ from ring A to form a fragment ion at *m*/*z* 125.04. Based on the above, the compound was presumed to be aromadendrin of the SJT-DB, and was further confirmed by comparison with the reference substance.

C116, C123, C120, and C129 are a group of isomers (C_21_H_22_O_10_). The quasi-molecular ion at *m*/*z* 433.11 produces the aglycone fragment ion at *m*/*z* 287.05 after the loss of glycosyl. Based on the literature [33], they were presumed to be engeletin and its isomers of the SJT-DB. C120 was confirmed to be engeletin by comparison with the reference substance.

##### 3.3.4.3 Identification of flavonoids and flavonols

These compounds are mostly present in the form of quercetin (C_15_H_10_O_7_, M = 302.04) or kaempferol (C_15_H_10_O_6_, M = 286.05) *O*-glycosides or *C*-glycosides in SJT. The *C*-glycosides of flavonoids are prone to saccharide ring cleavage, while the *O*-glycosides of flavonoids are prone to the loss of the whole saccharide moiety. Hence, flavonoid *C*-glycosides and *O*-glycosides can be distinguished according to the fragmentation characteristics of the saccharide moiety.

C139 showed [M−H]^−^ at *m*/*z* 301.03 in the MS^1^ spectra, and its fragment ions were consistent with the fragmentation profile of quercetin described in the literature (An et al., 2013). It was unequivocally identified as quercetin by comparison with the reference substance.

C157 showed [M−H]^−^ at *m*/*z* 285.0400 in the MS^1^ spectra and the fragment ions [M−H−CO]^−^ at *m*/*z* 257.04 and [M−H−2CO]^−^ at *m*/*z* 229.05 characteristic of kaempferol in the MS^2^ spectra. It was unequivocally identified as kaempferol by comparison with the reference substance.

C64, C82, and C110 showed M−H]^−^ at *m*/*z* 593.1523 in the MS^1^ spectra. C64 and C110 were identified as vicenin-2 and nicotflorin of the SJT-DB by comparison with the reference substances, with vicenin-2 being a flavonoid *C*-glycoside and nicotflorin a flavonoid *O*-glycoside. The fragmentation pathway of vicenin-2 is the same as that reported in the literature (Zhong et al., 2019). The MS^2^ spectra of nicotflorin showed the kaempferol ion at *m*/*z* 285.04 and the fragment characteristic of kaempferol at *m*/*z* 229.05. The MS^2^ spectra of C82 showed a fragment ion [M−H−C_4_H_8_O_4_]^−^ at *m*/*z* 473.1028 produced by glucose ring cleavage and a fragment ion at *m*/*z* 429.0716 produced by the loss of rhamnose; hence, C82 was presumed to be kaempferol 3-*O*-β-D-glucopyranosyl-7-*O*-α-L-rhamnopyranoside, a compound in the SJT-DB.

C102 showed [M−H]^−^ at *m*/*z* 739.2029 in the MS^1^ spectra, and fragment ions such as [M−H−Rha]^−^ at *m*/*z* 593.1447 and [M−H−Rha−Glc−H_2_O]^−^ at *m*/*z* 413.0943 can be seen in the MS^2^ spectra; hence, it was presumed to be acacetin 7-*O*-(6″-*O*-a-L-rhamnopyranosyl)-β-sophoroside, a compound in the SJT-DB.

C96 showed [M−H]^−^ at *m*/*z* 609.1487 in the MS^1^ spectra and was confirmed as rutin by consulting the literature (An et al., 2013) and in comparison with the reference substance.

C99 showed [M−H]^−^ at *m*/*z* 463.0884 in the MS^1^ spectra, and its molecular formula was presumed to be C_21_H_20_O_12_. The MS^2^ spectra showed fragment ions at *m*/*z* 301.0322, m/*z* 255.0314, m/*z* 225.0518, m/*z* 179.0010, and *m*/*z* 151.0067. Hence, C99 was presumed to be quercetin monoglycoside. The compound isoquercetin that matched the above data was found by consulting the SJT-DB. C99 was further confirmed as isoquercetin by consulting the literature (Wu et al., 2022) and in comparison with the reference substance.

C104 showed [M−H]^−^ at *m*/*z* 477.0675, which produced a fragment ion at *m*/*z* 301.03 by the loss of glucuronic acid. C104 was confirmed to be mikwelianin by comparison with the reference substance.

C106 showed [M−H]^−^ at *m*/*z* 577.1580 in the MS^1^ spectra and the fragment ions at *m*/*z* 431.0967 and *m*/*z* 285.039 formed by the continuous loss of rhamnose in the MS^2^ spectra. Its structure was presumed to be kaempferol linked with two rhamnosyl groups. C106 was further confirmed as kaempferitrin by comparison with the reference substance.

C109, C113, C114, and C117 are a group of isomers and showed the quasi-molecular ion [M−H]^−^ at *m*/*z* 447.09 in the MS^1^ spectra. The aglycone of C109, C113, and C114 can be identified as kaempferol and that of C117 as quercetin according to the fragment ions at *m*/*z* 301.03 and 285.04 in the MS^2^ spectra. C113, C114, and C117 were confirmed as kaempferol-7-*O*-β-D-glucopyranoside, kaemferol-3-*O*-β-D-glucopyranoside, and quercetin 3-*O*-α-L-rhamnopyranoside, respectively, by comparison with the reference substances, and C109 was presumed to be kaempferol-5-*O*-β-D-glucopyranoside, a compound in the SJT-DB.

C121 showed the ion [M−H]^−^ at *m*/*z* 477.1049, which produced a fragment ion at *m*/*z* 314.04 after the homolytic cleavage of glucosyl, and it was confirmed as isorhamnetin-3-*O*-β-D-glucoside, a compound in the SJT-DB by comparison with the reference substance.

C122 showed [M−H]^−^ at *m*/*z* 461.0746, and its molecular formula was predicted to be C_21_H_18_O_12_. The [M−H]^−^ at *m*/*z* 461.0746 lost glucuronyl to form the aglycon ion at *m*/*z* 285.0371, which lost one carbonyl from ring C to form a fragment ion at *m*/*z* 257.0453, which lost one CO molecule to generate a fragment ion at *m*/*z* 229.0480. The fragment ion at *m*/*z* 257.0453 can also lose a CO_2_ molecule to generate a fragment ion at *m*/*z* 213.0497 or lose a C_2_H_2_O_2_ molecule to produce a fragment ion at *m*/*z* 199.0503. Therefore, C122 was presumed to be kaempferol-3-*O*-β-D-glucuronide, a compound in the SJT-DB.

#### 3.3.5 Identification of pentacyclic triterpenoids

##### 3.3.5.1 Identification of triterpenes of oleanane and ursane types

Most of the pentacyclic triterpenoids in SJT are of the oleanane and ursane types, and they often exist in pairs, mainly derived from CH and RLR. The common fragmentation feature of these compounds is the loss of CO_2_, CO, and H_2_O, and the glycosidic bond is prone to breakage.

C140 and C141 are a pair of isomers; both showed [M−H]^−^ at *m*/*z* 973.50 in the MS^1^ spectra, and their molecular formula was predicted to be C_48_H_78_O_20_. The characteristic ion [M−H−2Glc−Rha]^−^ at *m*/*z* 503.34 and glycosyl fragment ions at *m*/*z* 469.16, m/*z* 367.12, and *m*/*z* 323.10 were present in the MS^2^ spectra. Hence, C140 and C141 were confirmed to be madecassoside and asiaticoside B, respectively, by consulting the literature (Long et al., 2012) and a comparison with the reference substances.

C143, C145, C146, C148, C154, C159, C162, C163, C165, C170, and C172 are a group of isomers (C_48_H_78_O_19_). C148 was confirmed to be asiaticoside by consulting the literature (Long et al., 2012) and a comparison with the reference substance. The aglycon ion [M−H−Rha−2Glc]^−^ at *m*/*z* 487.34 and the saccharide moiety ion at *m*/*z* 469.16 were seen in the MS^2^ spectra.

C160, C174, C175, C177, C179, and C180 are a group of isomers; they showed [M−H]^−^ at *m*/*z* 503.34 in the MS^1^ spectra, and their molecular formula was predicted to be C_30_H_48_O_6_. By comparison with the reference substances, C174 and C177 were confirmed to be 19α-hydroxyasiatic acid derived from RLR and madecassic acid derived from CH, respectively, and C160 and C175 with similar fragmentation behaviors were presumed to be their isomers. C179 and C180 showed different fragment ions in the MS^2^ spectra in comparison with the other isomers, and no [M−H−CO_2_]^−^ at *m*/*z* 459.35 was seen. The carboxyl group was presumed to be absent at position C-28. The characteristic fragment ion [M−H−CH_2_O−H_2_O]^−^ at *m*/*z* 437.31 was seen in the MS^2^ spectra, and the methanol group was presumed at position C-28. By consulting the SciFinder database, C179 and C180 were presumed to be camelliagenin E or its isomer.

C186, C189, C192, and C193 are a group of isomers (C_30_H_48_O_5_), and C192 and C193 were confirmed to be tormentic acid and euscaphic acid, respectively, by comparison with the reference substances. The loss of the carboxyl group at position C-28, dehydration, and ring A cleavage were their major fragmentation behaviors.

C176 and C178 are a pair of isomers (C_35_H_52_O_10_), and the fragment ions [M−H−C_6_H_10_O_5_]^−^ at *m*/*z* 469.30, [M−H−C_6_H_10_O_5_−H_2_O]^−^ at *m*/*z* 451.29, and [M−H−C_6_H_10_O_5_−H_2_O−CO_2_]^−^ at *m*/*z* 407.30 were seen in their MS^2^ spectra; thus, the glycosyl, hydroxyl, and carboxyl groups were presumed to be present. The structure of 24-noroleana-3,12-dien-28-oic acid, 3,15-dihydroxy-2-oxo-, *β*-D-glucopyranosyl ester was found to be consistent with the above fragments based on consultation with SciFinder. The other compound was presumed to be its isomer and also a potential new compound. The difference between the two compounds was presumed to lie in the two methyl sites of ring E. That is, they are of oleanane and ursane types, respectively.

##### 3.3.5.2 Identification of other pentacyclic triterpenes

C134, C135, C136, C138, C144, C150, C153, C155, C166, C169, C171, and C173 are a group of isomers; the [M−H]^−^ at *m*/*z* 711.40 can be seen in their MS^1^ spectra, and their molecular formula was predicted to be C_37_H_60_O_13_. Neutral losses of HCOOH, C_6_H_12_O_6_, C_7_H_12_O_7_, and H_2_O can be seen in their MS^2^ spectra. By consulting SciFinder, it was found that (2α,3β,4β,19α)-23-(glycero-manno-heptonoyloxy)-2,3,19 trihydroxyolean-12-en-28-oic acid and (2α,3β,4α,6β)-23-(glycero-manno-heptonoyloxy)-2,3,6-trihydroxyurs-12-en-28-oic acid are consistent with the fragment ions seen in the MS^2^ spectra. It was presumed that the other 10 compounds are their isomers and also potential new compounds. It was also presumed that the skeletons of the new compounds are the same as those of the above two compounds, but the binding sites of the carboxyl, hydroxyl, and glycosyl groups differ.

C187 and C188 are a pair of isomers, and characteristic neutral losses such as CO_2_, CH_2_O_2_, C_5_H_8_O_4_, and H_2_O can be seen in the MS^2^ spectra. Based on consultation of SciFinder, the side chains of esculentoside B were found to contain the above characteristic structure; hence, C187 and C188 were presumed to be esculentoside B and its isomer.

### 3.4 Construction of the “herb–compound–target” network

Based on the SuperPred and SwissTargetPrediction databases, a total of 254 targets were predicted for the 183 SJT compounds identified by MS in this paper (SJT-MS, 196 compounds minus 13 potential new compounds) and a total of 525 targets for 618 compounds in SJT-DB (Supplementary Table S2). Since the SJT-MS compounds are better at reflecting the actual composition of SJT, the targets for the SJT-MS compounds were used in the network pharmacology analysis.

**FIGURE 5:**
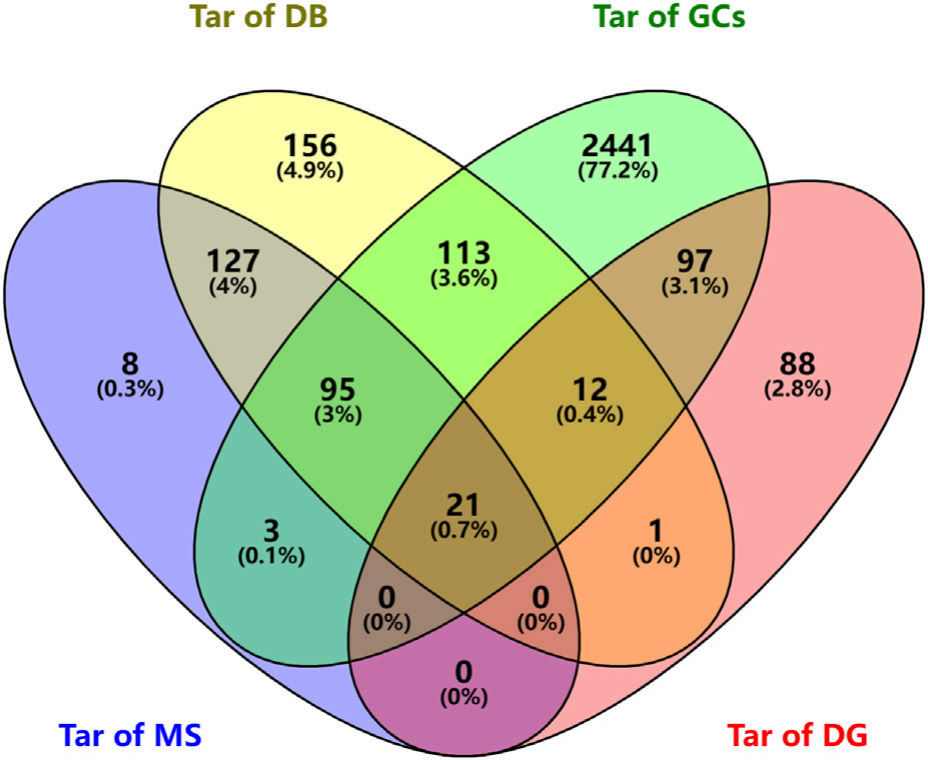
Venn diagram of predicted targets of SJT and UTIs (Tar of MS: Targets of compounds of SJT identified by MS; Tar of DB: Targets of compounds in SJT database; Tar of GCs: Targets of UTIs in GeneCards; Tar of DG: Targets of UTIs in DisGeNET).

**FIGURE 6:**
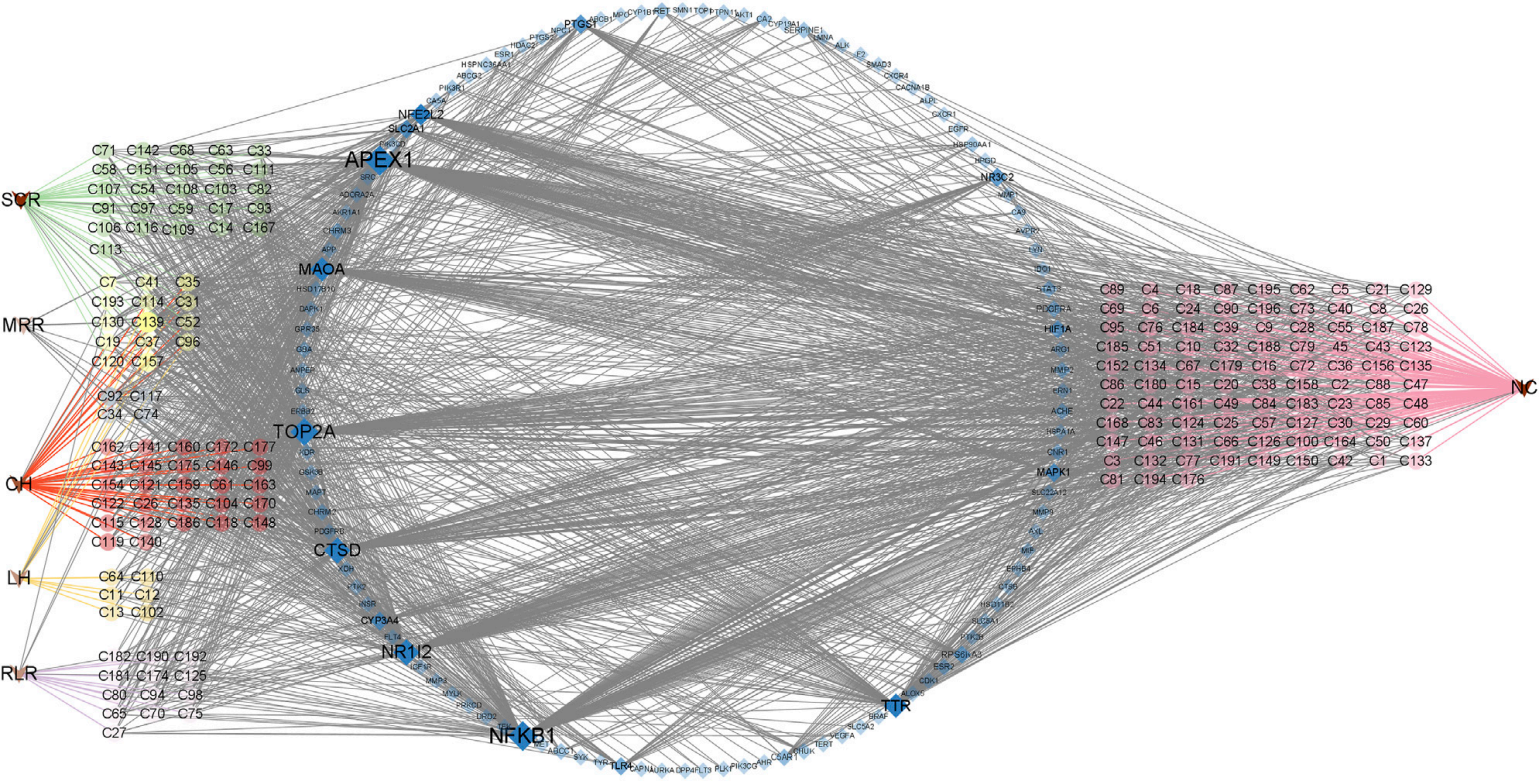
The “herb–compound–target” network of SJT. (V triangles indicate herbs; circles indicate the main constituents of different herbs; SCR, Smilacis Chinae Rhizoma; MRR, Melastoma Radix et Rhizoma; CH, Centellae Herba; LH, Lygodii Herba; RLR, Rosae Laevigatae Radix; NC indicates the compounds are not reported in the five herbs of SJT; blue diamonds indicate targets).

We searched the DisGeNET and GeneCards databases using “urinary tract infections” as the search term and found 219 and 2782 disease-related targets, respectively (Supplementary Table S2); coincident targets were identified by comparison with the targets for the SJT-MS compounds (Figure 5). A total of 119 common targets for disease (UTIs) and compounds were identified (Supplementary Table S2) and visualized using Cytoscape 3.9.1 software (Figure 6) to construct the “herb–compound–target” network.

### 3.5 PPI network analysis

The 119 common targets of diseases and compounds obtained in Section 3.4 were uploaded to the STRING 11.0 database (https://cn.string-db.org/, accessed on 4 January 2023). We set the protein type to “*H. sapiens*.” In order to ensure the reliability of the results, the minimum interaction score was set to 0.7, and a protein interaction network with 124 nodes and 523 edges was obtained. Then, the results were imported into Cytoscape software, and the CytoNCA plug-in was used to calculate the degree value of the network. A total of 20 core targets related to UTIs were selected from the highest degree to the lowest degree, as shown in Table 1.

**TABLE 1:** Top 20 core targets of the 183 compounds of SJT for treating UTIs.

Rank	Symbol	Protein names	Degree
1	HSP90AA1	Heat shock protein HSP 90-alpha	43.0
2	SRC	Proto-oncogene tyrosine-protein kinase Src	42.0
3	STAT3	Signal transducer and activator of transcription 3	38.0
4	AKT1	RAC-alpha serine/threonine-protein kinase	36.0
5	EGFR	Epidermal growth factor receptor	35.0
6	PIK3R1	Phosphatidylinositol 3-kinase regulatory subunit alpha	31.0
7	VEGFA	Vascular endothelial growth factor A	30.0
8	PTPN11	Tyrosine-protein phosphatase non-receptor type 11	30.0
9	MAPK1	Mitogen-activated protein kinase 1	29.0
10	PTK2	Tyrosine kinase 2	26.0
11	ESR1	Estrogen receptor	26.0
12	LYN	Tyrosine-protein kinase Lyn	22.0
13	PIK3CD	Phosphatidylinositol 4,5-bisphosphate 3-kinase catalytic subunit delta isoform	21.0
14	HIF1A	Hypoxia-inducible factor 1-alpha	20.0
15	ERBB2	Receptor tyrosine-protein kinase erbB-2	19.0
16	CXCR4	C-X-C chemokine receptor type 4	19.0
17	TLR4	Toll-like receptor 4	19.0
18	MMP9	Matrix metalloproteinase-9	19.0
19	IGF1R	Insulin-like growth factor 1 receptor	17.0
20	CCND1	G1/S-specific cyclin-D1	17.0

### 3.6 GO and KEGG enrichment analyses of core targets

**FIGURE 7:**
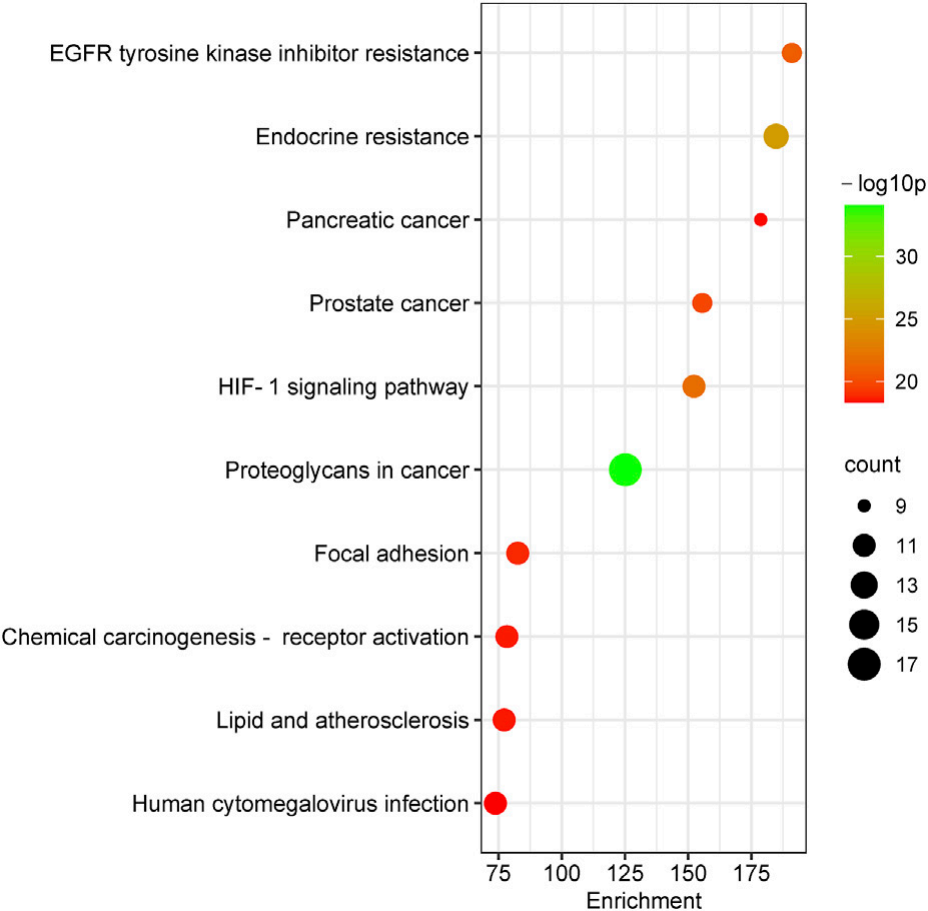
The major enrichment analyses of the KEGG pathways of SJT.

**FIGURE 8:**
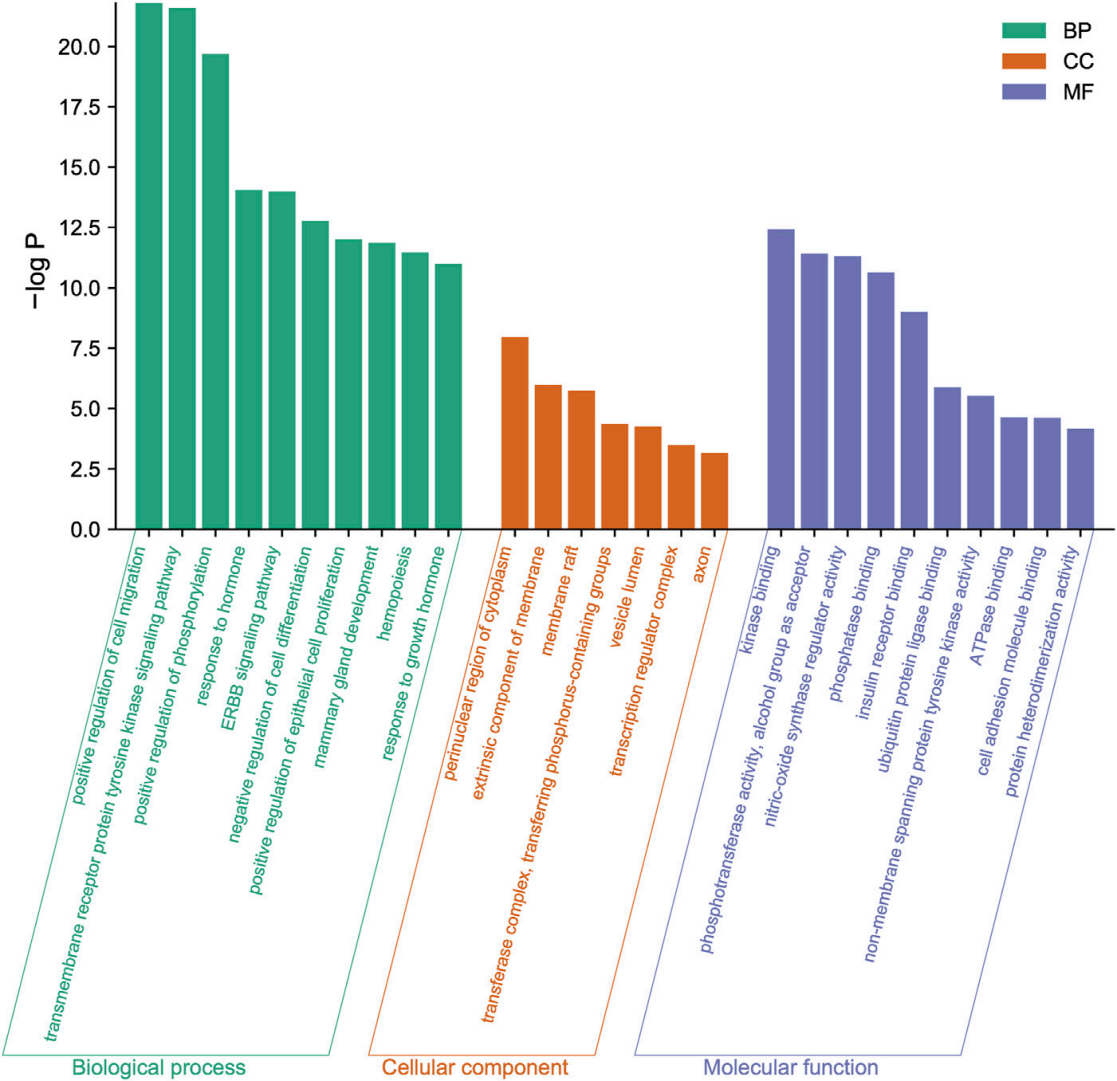
The major enrichment analyses of GO terms of SJT include biological process (BP), cellular component (CC), and molecular function (MF).

To further reveal the related pathways and mechanisms of SJT in the treatment of UTIs, we imported the 20 core targets into the Metascape database for GO and KEGG enrichment analyses. The top 10 KEGG signaling pathways related to UTIs were constructed based on a *p*-value <0.01, a minimum count of three, and an enrichment factor of >1.5, in the descending order of the enrichment factors (Figure 7), which are primarily associated with EGFR tyrosine kinase inhibitor resistance (hsa01521), endocrine resistance (hsa01522), pancreatic cancer (hsa05212), prostate cancer (hsa05215), the HIF-1 signaling pathway (hsa04066) (Lin et al., 2015), and proteoglycans in cancer (hsa05205). Similarly, the major enrichment analyses of the GO signaling pathways (GO-MF; GO-BP; GO-CC) were constructed based on a *p*-value <0.01, a minimum count of three, and an enrichment factor of >1.5, in the descending order of the enrichment factors (Figure 8). GO analysis indicated that these overlapping genes were associated with various biological functions. Among them, biological processes (BP) include the positive regulation of cell migration (GO:0030335), the transmembrane receptor protein tyrosine kinase signaling pathway (GO:0007169), positive regulation of phosphorylation (GO:0042327), response to hormone (GO:0009725), and the EGF receptor family signaling pathway (GO:0038127), etc.,; cellular component (CC) includes the perinuclear region of cytoplasm (GO:0048471), the extrinsic component of membrane (GO:0019898), membrane raft (GO:0045121), transferase complex, transferring phosphorus-containing groups (GO:0061695), vesicle lumen (GO:0031983), etc.,; and molecular function (MF) includes kinase binding (GO:0019900), phosphotransferase activity, alcohol group as acceptor (GO:0016773), nitric-oxide synthase regulator activity (GO:0030235), phosphatase binding (GO:0019902), insulin receptor binding (GO:0005158), etc.

### 3.7 Construction of “potential effective compound–core target–signaling pathway” network

**FIGURE 9:**
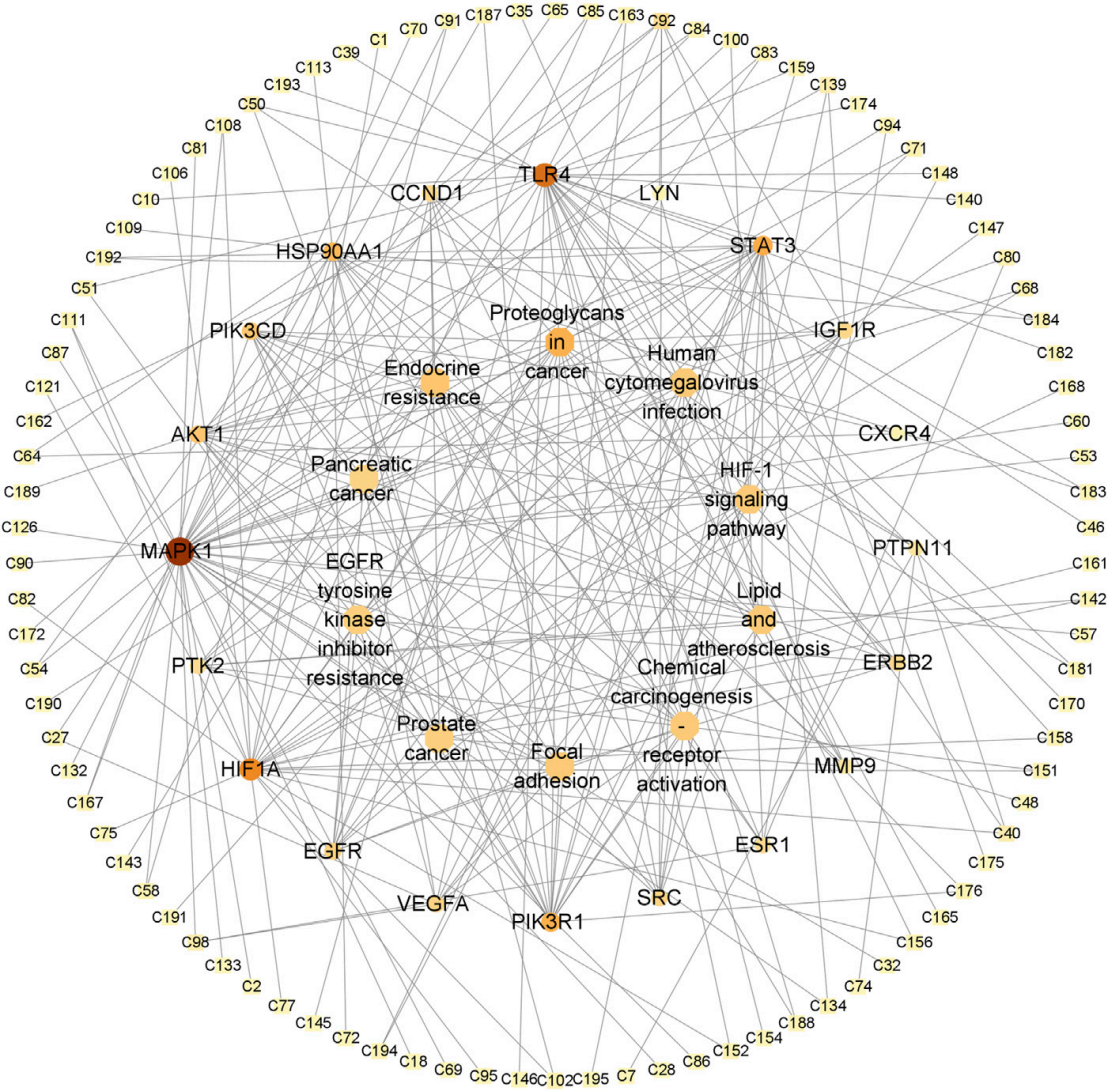
The “potential effective compound–core target–signaling pathway” network of SJT.

By integrating the core targets in Section 3.5 with the KEGG signaling pathways in Section 3.6 and target–compound relationships in Section 3.4, 94 potential effective compounds related to UTIs were obtained, acting on 20 core targets and playing therapeutic roles by regulating 10 related signaling pathways (Supplementary Table S3), as shown in the figure (Figure 9). There are 124 nodes (94 potential effective compounds, 20 core targets, and 10 pathways), with a total of 256 edges in the network, which fully demonstrates the multicomponent–multitarget–multipathway characteristics of SJT in the treatment of UTIs.

### 3.8 Screening of the key effective substances

Based on the etiology and pathogenesis of UTIs, antimicrobial and anti-inflammatory drugs are mainly used in clinical treatment (Carey et al., 2020). A literature search found that 27 of the 183 known compounds of SJT-MS (Supplementary Table S4) have antimicrobial or anti-inflammatory activity; hence, we considered these 27 compounds as effective substances of SJT.

Among 94 potential effective compounds, 12 overlapped with the above-mentioned 27 effective substances of SJT. Six (procyanidin B3, pubescenoside A, asiaticoside, euscaphic acid, esculentoside B, vicenin-2) have anti-inflammatory activity only, and six (quercetin, malic acid, kaempferitrin, ellagic acid, caffeic acid, gallic acid) have both antimicrobial and anti-inflammatory activities. Therefore, these 12 effective substances were determined as key effective substances of SJT, and except for asiaticoside, caffeic acid, gallic acid, and quercetin, the other eight compounds were newly discovered in SJT.

### 3.9 Molecular docking verification results

**FIGURE 10:**
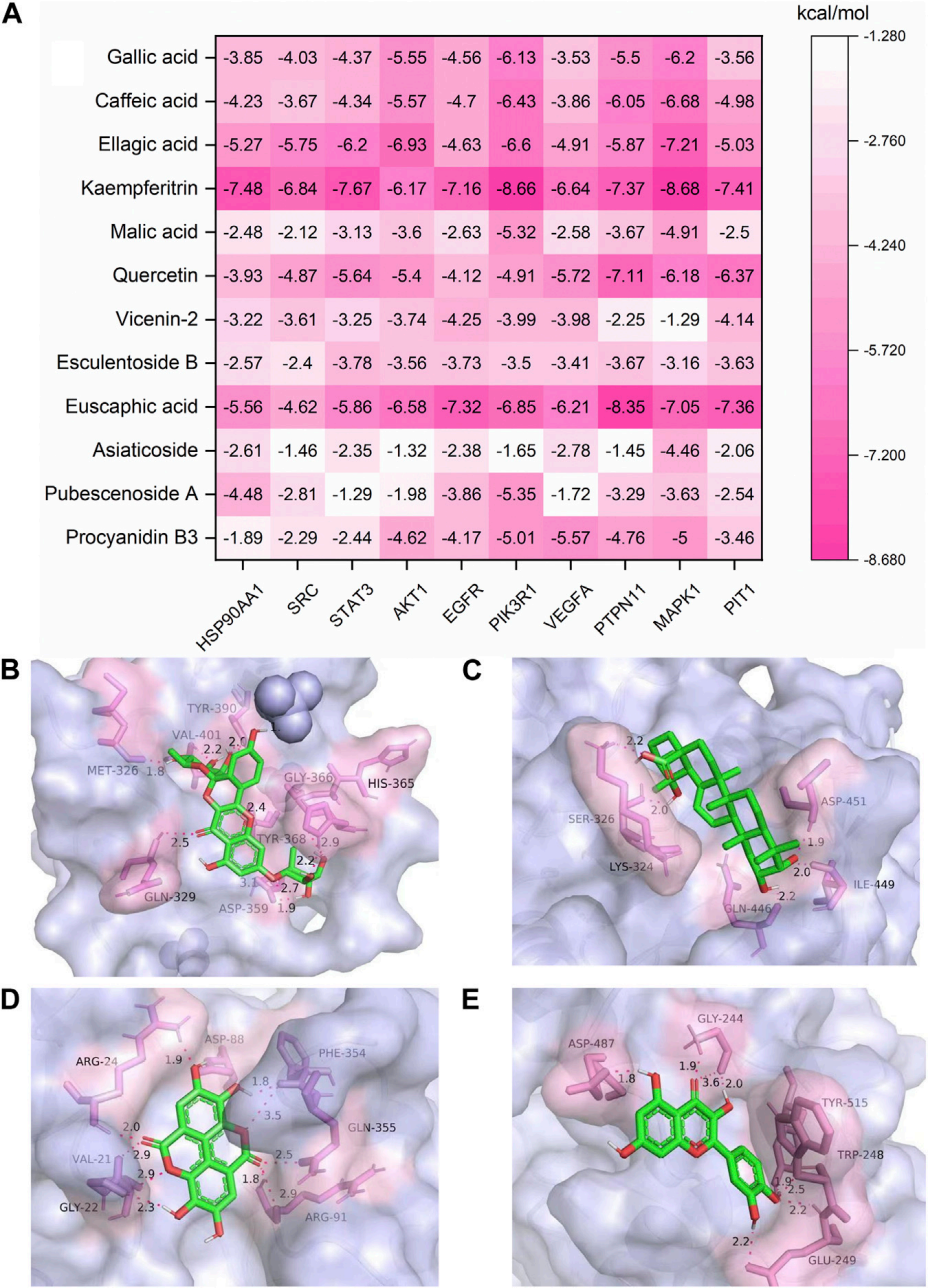
Molecular docking results of the 12 key effective substances with 10 core targets **(A)** and the binding interaction patterns of kaempferitrin with PIK3R1 **(B)**, euscaphic acid with PTPN11 **(C)**, ellagic acid with MAPK1 **(D)**, and quercetin with PTPN11 **(E)**.

In order to explore the binding force of these 12 key effective substances to key targets, we selected 10 key targets for molecular docking studies. Molecular docking software can be used not only to identify the correct conformation of ligands bound to active pockets of target proteins but also to estimate the strength of the interaction between target proteins and ligands. When the binding energy is < 0 kcal/mol, the small molecule ligand can spontaneously bind to the protein receptor. If the binding energy is < −1.2 kcal/mol or lower, it indicates that the two have a better binding ability (Wang et al., 2021). It was reported that PIT1 is a key target of UTIs; silencing PIT1 prevents and reduces acute UPEC infection in mouse bladders (Pang et al., 2022). The top nine potential targets in the PPI network and PIT1 were selected for molecular docking with 12 key effective substances, and the results are shown in Figure 10A. All 120 “compound–target” binding pairs were able to bond by hydrogen bonds, with binding energies of < −1.2 kcal/mol, among which kaempferitrin, euscaphic acid, ellagic acid, and quercetin showed strong binding activity with the 10 targets. The most stable binding energy of the four compounds was kaempferitrin with PIK3R1 (Figure 10B), euscaphic acid with PTPN11 (Figure 10C), ellagic acid with MAPK1 (Figure 10D), and quercetin with PTPN11 (Figure 10E). The specific binding patterns were processed and optimized by PyMoL software. In conclusion, the molecular docking results showed that the 12 key effective substances, such as kaempferitrin, euscaphic acid, and ellagic acid, had good binding force with the 10 key targets, such as PIK3R1.

## 4 Discussion

We identified 196 compounds from SJT in total, i.e., 13 potential new compounds and 183 known compounds, which could be found in SciFinder; 169 of the 183 known compounds were the first discovered constituents of STJ. Among the 183 known com-pounds, 90 were reported in the five constituent herbs included in the SJT-DB and their origins have been confirmed (16 from RLR, 38 from SCR, 8 from MRR, 12 from LH, 39 from CH), while the other 93 were not reported in the five herbs of SJT. Of the 196 compounds, 44 were unequivocally identified by comparison with the reference compounds, and 152 were preliminarily identified by consulting the MS data, the literature, and the SJT-DB (see Supplementary Figures S1–S225 for the LC-MS spectra and fragmentation pathways of the 196 compounds and 44 reference compounds). The peak area in the extracted ion chromatogram from 196 compounds was calculated in Supplementary Table S5. The top 20 compounds in terms of peak area included 10 organic acids, 9 flavonoids and 1 pentacyclic triterpene, and 10 of them were unequivocally identified by comparison with the reference compounds, including 1,3-dicaffeoylquinic Acid, 1,5-dicaffeoylquinic Acid, isochlorogenic acid C, neoastilbin, nicotflorin, chlorogenic acid, isochlorogenic acid B, isoastilbin, astilbin, neochlorogenic acid. This is the first study to fully demonstrate the chemical constituents of SJT and lays a foundation for further investigation of their effective substances.

SJT are made from a decoction of five Chinese herbs in the prescription, and the types of chemical constituents may change during the preparation process (e.g., transformation of thermally unstable compounds), so a network pharmacology study using SJT-MS compounds rather than SJT-DB compounds can more truly reflect the potential effective compounds and functional mechanism of SJT. Through the construction of PPI network diagrams, the core targets of SJT for the treatment of UTIs were screened, including HSP90AA1, SRC, STAT3, AKT1, EGFR, PIK3R1, VEGFA, PTPN11, MAPK1, HIF1A, TLR4, etc. A study has shown that HIF1A transcriptional regulation plays a key role in defense of the urinary tract against UPEC infection [38]. AKT1 is a key reg-ulator of host cell survival, inflammatory responses, proliferation, and metabolism (Wiles et al., 2008). It was reported that SRC could inhibit TLR4-induced inflammatory cytokines and promote the anti-inflammatory cytokine IL-10, which plays an important role in the treatment of acute and chronic inflammation (Li et al., 2019). Wittmann et al. (Wittmann et al., 2015) found that the increased expression of TLRs in patients might result in counter regulation of the host and serve to prevent intestinal inflammation and disease. In addition to the 20 core targets obtained by the PPI network, it was shown that the silencing of PIT1 prevents and reduces acute UPEC infection in mouse bladders.

Based on the target–compound relationships analysis, among the 183 known compounds, 94 compounds were found to act on 20 core targets related to UTIs and are regarded as potential effective compounds related to UTIs.

Considering that SJT can exert efficacy through antibacterial and anti-inflammatory effects, according to the literature review, 27 SJT-MS compounds (C1, C7, C19, C27, C31, C35, C37, C41, C57, C59, C64, C92, C93, C96, C97, C105, C106, C115, C120, C122, C139, C148, C157, C177, C186, C187, and C193) were found to possess antimicrobial and anti-inflammatory activities and are regarded as effective substances: organic acids, flavonoids, and triterpenoids in five herbs. Moreover, 20 of the 27 effective substances were first discovered in SJT.

Among the 27 effective substances, 12 effective substances (C1, C7, C27, C35, C57, C64, C92, C106, C139, C148, C187, and C193) overlapped with the above-mentioned 94 potential effective compounds—namely, they had both the related activities of anti-bacterial and anti-inflammatory effects and acted on the core targets related to UTIs, so they are regarded as key effective substances of SJT. It is noteworthy that, unlike conventional antibiotics, the active constituents of Chinese herbs can exert antimicrobial activity through synergism (Li et al., 2022) and also have inhibitory effects on antibiotic-resistant bacteria (Su et al., 2020). For example, quercetin (C139) was shown to inhibit SRC tyrosine phosphorylation and its kinase activity, thereby limiting the LPS-induced inflammatory response (Endale et al., 2013). In addition, quercetin (C139) is often used in combination with other antimicrobial agents to reverse the antibiotic resistance of bacteria (Pal and Tripathi, 2019; Nguyen and Bhattacharya, 2022). Rutin (C96) in SJT does not have significant antimicrobial activity, but it enhances the antimicrobial activity of quercetin (Arima et al., 2002).

The KEGG pathway enrichment analysis showed that EGFR tyrosine kinase inhibitor resistance, endocrine resistance, pancreatic cancer, prostate cancer, the HIF-1 signaling pathway, and proteoglycans in cancer and other pathways may be potential signaling pathways for the treatment of UTIs with SJT. It has been reported that the HIF-1 signaling pathway is an important mechanism in the treatment of UTIs and that HIF-1α transcriptional regulation plays a key role in the defense of the urinary tract against UPEC infection (Lin et al., 2015), whereas quercetin acts on EGFR (Huang et al., 2009), AKT1 (Spencer et al., 2003), IGF1R (El et al., 2014), and CAMK2B (Wang et al., 2011) targets in the HIF-1 signaling pathway; ellagic acid (C92) acts therapeutically on EGFR, AKT1, IGF1R, and ERBB2 targets in the HIF-1 signaling pathway (Hundsdorfer et al., 2012). These findings indicate that SJT are likely to act on the HIF-1 signaling pathway as well as the above-mentioned targets and exert their effect. Also, SJT is likely to act on those pathways above-mentioned.

The molecular docking results showed that the 12 key effective substances and 10 core targets had good docking results, with an average binding energy of −4.53 kcal/mol. Studies have shown that PIT1 is a key target in the treatment of UTIs (Pang et al., 2022). The average binding energy of the 12 key effective substances with PIT1 was −4.42 kcal/mol. Therefore, PIT1 is regarded as the core target of SJT in the treatment of UTIs. The average binding energy of the five other targets, namely, MAPK1, PIK3R1, PTPN11, AKT1, and EGFR, was lower than that of PIT1, suggesting that they may be the key targets of SJT in the treatment of UTIs.

## 5 Conclusion

A total of 196 compounds in SJT, i.e., 80 organic acids, 9 phenols, 58 flavonoids, and 49 triterpenoids, were identified by analysis of LC-MS^n^ data, consulting the literature, and comparison with 44 reference compounds. Of the 196 compounds, 183 were known compounds and 13 were potential new compounds. Among the 183 known compounds, 169 are newly discovered constituents of SJT, and 93 were not reported in their five constituent herbs. Based on the network pharmacology results, 94 of the 183 known compounds were predicted to act on 20 core targets and were considered potential effective compounds of SJT. According to the literature, 27 of the 183 known compounds were found to have antibacterial and anti-inflammatory activities and thus were verified as effective substances of SJT. Meanwhile, 12 of the 27 effective substances overlapped with the 94 potential effective compounds and could be considered key effective substances of SJT: malic acid, gallic acid, procyanidin B3, caffeic acid, pubescenoside A, ellagic acid, kaempferitrin, quercetin, asiaticoside, esculentoside B, euscaphic acid, and vicenin-2. A total of 21 core targets were considered to be associated with UTIs; 20 targets, i.e., HSP90AA1, SRC, STAT3, AKT1, EGFR, PIK3R1, VEGFA, PTPN11, MAPK1, PTK2, ESR1, LYN PIK3CD, HIF1A, ERBB2, CXCR4, TLR4, MMP9, IGF1R, and CCND1, were obtained by network pharmacology, while one remaining target, PIT1, was obtained by literature retrieval. SJT may mitigate the inflammatory response of the host to infection by acting on the HIF-1 signaling pathway, prostate cancer, and other signaling pathways. Finally, the 12 key effective substances were verified to bind with 10 core targets using the molecular docking technique. In conclusion, this study clarified the chemical constituents, effective substances, core targets, and functional mechanism of SJT and provided a solid foundation for understanding the effective substances and mechanism of SJT.

## Data Availability

The datasets presented in this study can be found in online repositories. The names of the repository/repositories and accession number(s) can be found in the article/Supplementary Material.
