# Supplementary material for: Chemical profiling of Sanjin tablets and exploration of their effective substances and mechanism in the treatment of urinary tract infections
Source: Front Chem. 2023 Jun 20;11:1179956. doi: 10.3389/fchem.2023.1179956 (PMC10318440; doi:10.3389/fchem.2023.1179956)
Supplement: Supplementary file 6 [file DataSheet1.docx]

Supplementary Material

Chemical Profiling of Sanjin Tablets and Exploration of Their Effective Substances and Mechanism in the Treatment of Urinary Tract Infections

**Meng-Yuan Li ^1,2^, Yang Li ^2^, Li-Li Wang ^1*^, Feng Xu ^2*^, Xu-Yan Guo ^2^, Jing Zhang ^2^, Yang Lv ^2^, Peng-Pu Wang ^2^, Shun-Qi Wang ^2^, Jian-Guo Min ^3^, Xun Zou ^3*^, and Shao-Qing Cai ^2^**

^1^School of Pharmacy, Henan University of Chinese Medicine, Zhengzhou, China

^2^School of Pharmaceutical Sciences, Peking University, Beijing, China

^3^Guilin Sanjin Pharmaceutical Company Limited, Guilin, China

*** Correspondence:** Li-Li Wang: wllywlly2004@163.com; Feng Xu: xufeng_pharm@163.com;

Xun Zou: xunzou@hotmail.com

# Proposed fragmentation pathways for 196 compounds

C1: Malic acid

Malic acid

**Figure S1.** Extracted ion chromatogram (EIC) of *m/z* 133.01 detected in negative ion detection mode

**Figure S2.** Proposed fragmentation pathway of C1 (Malic acid)

C2: 2-Butenedioic acid (2Z)-, 1-[1-carboxy-3-(hydroxymethoxy)-3-oxopropyl] 4-(hydroxymethyl) ester

2-Butenedioic acid (2Z)-, 1-[1-carboxy-3-(hydroxymethoxy)-3-oxopropyl] 4-(hydroxymethyl) ester

**Figure S3.** Extracted ion chromatogram (EIC) of *m/z* 291.03 detected in negative ion detection mode

**Figure S4.** Proposed fragmentation pathway of C2 (2-Butenedioic acid (2Z)-, 1-[1-carboxy-3-(hydroxymethoxy)-3-oxopropyl] 4-(hydroxymethyl) ester)

C3, C4, C5, C6, C8, C9, C10: Gallic glucoside or isomer

Gallic glucoside 1*-O-*Galloyl-α-D-glucopyranose

3-(α-D-Glucopyranosyloxy)-4,5-dihydroxybenzoic acid 3-*O*-Galloyl-β-D-glucopyranose

4-(α-D-Glucopyranosyloxy)-3,5-dihydroxybenzoic acid

4-(β-D-Glucopyranosyloxy)-3,5-dihydroxybenzoic acid Gallic acid 3*-O-*β-glucoside

**Figure S5.** Extracted ion chromatogram (EIC) of *m/z* 331.05 detected in negative ion detection mode

**Figure S6.** Proposed fragmentation pathway of C3, C4, C5, C8, C9 or C10 (Gallic glucoside or isomer)

C7: Gallic acid

Gallic acid^a(a: Indicates that the compound is a chemical component in the Sanjin Tablet compound database(SJT-DB), the same below)^

**Figure S7.** Extracted ion chromatogram (EIC) of Gallic acid detected in negative ion detection mode

**Figure S8.** Proposed fragmentation pathway of C7 (Gallic acid)

C11, C12, C13, C16, C20, C22, C23, C25, C30: 6*-O-*Caffeoyl*-*β*-*D-glucopyranose or isomer

6*-O-*Caffeoyl*-*β*-*D-glucopyranose^a^ 3*-O-*Caffeoyl*-*β*-*D-glucopyranose^a^

4*-O-*Caffeoyl*-*β*-*D-glucopyranose^a^ 3*-O-*Caffeoyl*-*α*-*D-glucopyranose

4*-O-*Caffeoyl*-*α*-*D-glucopyranose 6*-O-*Caffeoyl*-*α*-*D-glucopyranose

1*-O-*Caffeoyl*-*α*-*D-glucopyranose 1*-O-*Caffeoyl*-*β*-*D-glucopyranose

2*-O-*Caffeoyl*-*α*-*D-glucopyranose

**Figure S9.** Extracted ion chromatogram (EIC) of *m/z* 341.09 detected in negative ion detection mode

**Figure S10.** Proposed fragmentation pathway of C11, C12, C13, C16, C20, C22, C23, C25 or C30 (6*-O-*Caffeoyl*-*β*-*D-glucopyranose or isomer)

C14, C29: Myrciaphenone A or isomer

Myrciaphenone A^a^ 1-[2,4-Dihydroxy-6-(α-D-mannopyranosyloxy)phenyl]ethanone

**Figure S11.** Extracted ion chromatogram (EIC) of *m/z* 329.09 detected in negative ion detection mode

**Figure S12.** Proposed fragmentation pathway of C14 or C29 (Myrciaphenone A or isomer)

C15, C19, C37, C41: Isochlorogenic acid, Neochlorogenic acid, Chlorogenic acid, Cryptochlorogenic acid

Isochlorogenic acid Neochlorogenic acid^a^ Chlorogenic acid^a^

Cryptochlorogenic acid^a^

**Figure S13.** Extracted ion chromatogram (EIC) of *m/z* 353.09 detected in negative ion detection mode

**Figure S14.** Proposed fragmentation pathway of C15, C19, C37 or C41 (Chlorogenic acid or isomer)

C17: Methyl 2-(*β-D*-glucopyranosyloxy)-4,6-dihydroxybenzoate

Methyl 2-(*β-D*-glucopyranosyloxy)-4,6-dihydroxybenzoate^a^

**Figure S15.** Extracted ion chromatogram (EIC) of Methyl 2-(β*-*D-glucopyranosyloxy)-4,6-dihydroxybenzoate detected in negative ion detection mode

**Figure S16.** Proposed fragmentation pathway of C17 (Methyl 2-(β*-*D-glucopyranosyloxy)-4,6-dihydroxybenzoate)

C18, C21, C24, C26, C28, C32, C36, C38, C44, C48: 1*-O-p-*Coumaroyl*-β-D*-glucose or isomer

1*-O-p-*Coumaroyl*-*β*-*D-glucose Glucosido-*p*-coumaric acid

*cis*-Melilotoside Melilotoside

6*-O-p*-Coumaroyl-D-glucopyranoside 4*-O-*Coumaroylglucose

3-[4-(D-Galactopyranosyloxy)phenyl]-2-propenoic acid

3-[3-(β-D-Glucopyranosyloxy)phenyl]-2-propenoic acid D-Glucose, 1-m-hydroxycinnamate

Cinnamic acid, p-hydroxy-, 3-ester with D-glucopyranose

**Figure S17.** Extracted ion chromatogram (EIC) of *m/z* 325.09 detected in negative ion detection mode

**Figure S18** Two proposed fragmentation pathways of C18, C21, C24, C26, C28, C32, C36, C38, C44, C48 (1*-O-p-*Coumaroyl*-*β*-*D-glucose or isomer)

C27: Procyanidin B3

Procyanidin B3^a^

**Figure S19.** Extracted ion chromatogram (EIC) of *m/z* 577.14 detected in negative ion detection mode

**Figure S20.** Proposed fragmentation pathway of C27 (Procyanidin B3)

C31, C52: (+)-Catechin, (-)-Epicatechin

(+)-Catechin^a^ (-)-Epicatechin^a^

**Figure S21.** Extracted ion chromatogram (EIC) of *m/z* 289.07 detected in negative ion detection mode

**Figure S22.** Proposed fragmentation pathway of C31 ((+)-Catechin)

C33, C43, C72: (2R,3R)-Taxifolin 3*-O-*β*-*D-glucopyranoside or isomer

(2R,3R)-Taxifolin 3*-O-*β*-*D-glucopyranoside^a^ (2S,3S)-Taxifolin 3*-O-*β*-*D-glucopyranoside

Taxifolin 7*-O-*β-D-glucoside

**Figure S23.** Extracted ion chromatogram (EIC) of *m/z* 465.10 detected in negative ion detection mode

**Figure S24.** Proposed fragmentation pathway of C33, C43 or C72 ((2R,3R)-Taxifolin 3*-O-*β*-*D-glucopyranoside or isomer)

C34, C46, C50: 4-Hydroxy-3-methoxyphenyl-1*-O-*(6'*-O-*galloyl)*-*β*-*D-glucopyranoside) or isomer

4-Hydroxy-3-methoxyphenyl-1*-O-*(6'*-O-*galloyl)*-*β*-*D-glucopyranoside)^a^

4-Hydroxy-2-methoxyphenol-1*-O-*β*-*D-(6'*-O-*galloyl)glucopyranoside)

1*-O-*β-D-(6'*-O-*Galloyl)glucuropyranosyl-3-methoxy-5-hydroxybenzene

**Figure S25.** Extracted ion chromatogram (EIC) of *m/z* 291.03 detected in negative ion detection mode

**Figure S26.** Proposed fragmentation pathway of C34, C46 or C50 (4-Hydroxy-3-methoxyphenyl-1*-O-*(6'*-O-*galloyl)*-*β*-*D-glucopyranoside) or isomer)

C35: Caffeic acid

Caffeic acid^a^

**Figure S27.** Extracted ion chromatogram (EIC) of Caffeic Acid detected in negative ion detection mode

**Figure S28.** Proposed fragmentation pathway of C35 (Caffeic Acid)

C39, C51: (3,5-Dimethoxy-4-hydroxyphenyl)-1*-O-*β*-*D-(6*-O-*galloyl)glucopyranoside or isomer

(3,5-Dimethoxy-4-hydroxyphenyl)-1*-O-*β*-*D-(6*-O-*galloyl)glucopyranoside

(4,5-Dimethoxy-3-hydroxyphenyl)-1*-O-*β*-*D-(6*-O-*galloyl)glucopyranoside

**Figure S29.** Extracted ion chromatogram (EIC) of *m/z* 483.11 detected in negative ion detection mode

**Figure S30.** Proposed fragmentation pathway of C39 or C51 ((3,5-Dimethoxy-4-hydroxyphenyl)-1*-O-*β*-*D-(6*-O-*galloyl)glucopyranoside or isomer)

C40: 3,5-Dimethoxy-4-[[6*-O-*(3,4,5-trihydroxybenzoyl)*-β-D*-glucopyranosyl]oxy]benzoic acid

3,5-Dimethoxy-4-[[6*-O-*(3,4,5-trihydroxybenzoyl)*-*β*-*D-glucopyranosyl]oxy]benzoic acid

**Figure S31.** Extracted ion chromatogram (EIC) of *m/z* *m/z* 511.11 detected in negative ion detection mode

**Figure S32.** Proposed fragmentation pathway of C40 (3,5-Dimethoxy-4-[[6*-O-*(3,4,5-trihydroxybenzoyl)*-*β*-*D-glucopyranosyl]oxy]benzoic acid)

C42, C55, C125: Phloridzin or isomer

Phloridzin^a^

1-[2-(β-D-Allopyranosyloxy)-4,6-dihydroxyphenyl]-3-(4-hydroxyphenyl)-1-propanone

1-[2-(α-D-Allopyranosyloxy)-4,6-dihydroxyphenyl]-3-(4-hydroxyphenyl)-1-propanone

**Figure S33.** Extracted ion chromatogram (EIC) of *m/z* 435.13 detected in negative ion detection mode

**Figure S34.** Proposed fragmentation pathway of C125 (Phloridzin)

C45: Decumbeside A

Decumbeside A

**Figure S35.** Extracted ion chromatogram (EIC) of *m/z* 491.15 detected in negative ion detection mode

**Figure S36.** Proposed fragmentation pathway of C45 (Decumbeside A)

C47: Gallic acid 3*-O-*(6'*-O-*galloyl)-β-D-glucopyranoside

Gallic acid 3*-O-*(6'*-O-*galloyl)-β-D-glucopyranoside

**Figure S37.** Extracted ion chromatogram (EIC) of *m/z* 483.07 detected in negative ion detection mode

**Figure S38.** Proposed fragmentation pathway of C47 (Gallic acid 3*-O-*(6'*-O-*galloyl)-β-D-glucopyranoside)

C49: 2,3,4,5,6,7-Hexahydroxy-9H-xanthen-9-one

2,3,4,5,6,7-Hexahydroxy-9H-xanthen-9-one

**Figure S39.** Extracted ion chromatogram (EIC) of *m/z* 291.03 detected in negative ion detection mode

**Figure S40.** Proposed fragmentation pathway of C49 (2,3,4,5,6,7-Hexahydroxyxanthone)

C53, C59, C67: 5*-O-*Caffeoylshikimic acid and isomer

5*-O-*Caffeoylshikimic acid^a^ 3*-O-*Caffeoylshikimic acid

4*-O-*Caffeoylshikimic acid

**Figure S41.** Extracted ion chromatogram (EIC) of *m/z* 335.07 detected in negative ion detection mode

**Figure S42.** Proposed fragmentation pathway of C59, C59 or C67 (5*-O-*Caffeoylshikimic acid or isomer)

C54: Eriodictyol 3'*-O-*β-D-glucoside

C56, C58: Arthromerin B and Helicioside A

Eriodictyol 3'*-O-*β*-*D-glucoside^a^

Arthromerin B^a^ Helicioside A^a^

**Figure S43.** Extracted ion chromatogram (EIC) of *m/z* 449.11 detected in negative ion detection mode

**Figure S44.** Proposed fragmentation pathway of C54 (Eriodictyol 3'*-O-*β*-*D-glucoside)

**Figure S45.** Proposed fragmentation pathway of C56 or C58 (Arthromerin B or isomer)

C57, C60: Pubescenoside A and Pubescenoside B

Pubescenoside A Pubescenoside B

**Figure S46.** Extracted ion chromatogram (EIC) of *m/z* 441.14 detected in negative ion detection mode

**Figure S47.** Proposed fragmentation pathway of C57 or C60 (Pubescenoside A or isomer)

C61, C112, C115, C118, C119, C128: Isochlorogenic acid A and isomer

1,3-Dicaffeoylquinic acid^a^ Isochlorogenic acid B^a^

Isochlorogenic acid A^a^ 1,4-Dicaffeoylquinic acid^a^

1,5-Dicaffeoylquinic acid^a^ Isochlorogenic acid C^a^

**Figure S48.** Extracted ion chromatogram (EIC) of *m/z* 515.12detected in negative ion detection mode

**Figure S49.** Proposed fragmentation pathway of C115, C61, C112, C115, C118, C119 or C128 (Isochlorogenic acid A or isomer)

C62, C69, C73, C76, C78, C79, C86, C88, C89, C101: Rotundarpenoside B or isomer

Rotundarpenoside B Oblongaroside B

(2E)-2-Methyl-but-2-ene-1,4-diol-6'-Ocaffeoyl-1*-O-*β-glucopyranoside

Hymenoside V Rotundarpenoside A

β-​D-​Glucopyranoside, (2E)​-​4-​hydroxy-​3-​methyl-​2-​buten-​1-​yl, 6-​[(2E)​-​3-​(3,​4-​dihydroxyphenyl)​-​2-​propenoate]

2-Propenoic acid, 3-(3,4-dihydroxyphenyl)-, 6'-ester with 4-(β-D-glucopyranosyloxy)-2-pentanone, [S-(E)]-

2-​Propenoic acid, 3-​[4-​[[4-​(β-​D-​glucopyranosyloxy)​-​3-​methyl-​2-​butenyl]​oxy]​-​3-​hydroxyphenyl]​-​, (E,​E)​- (9CI)

**Figure S50.** Extracted ion chromatogram (EIC) of *m/z* 425.14 detected in negative ion detection mode

**Figure S51.** Proposed fragmentation pathway of C62, C69, C73, C76, C78, C79, C86, C88, C89 or C101 (Rotundarpenoside B or isomer)

C63: 3,5,7,3',5'-Pentahydroxy-2R,3R-flavanonol 3*-O-*α-L-rhamnopyranoside

3,5,7,3',5'-Pentahydroxy-2R,3R-flavanonol 3*-O-*α-L-rhamnopyranoside^a^

**Figure S52.** Extracted ion chromatogram (EIC) of *m/z* 449.11 detected in negative ion detection mode

**Figure S53.** Proposed fragmentation pathway of C63 (3,5,7,3',5'-Pentahydroxy-2R,3R-flavanonol 3*-O-*α-L-rhamnopyranoside)

C64: Vicenin-2

Vicenin-2^a^

**Figure S54.** Extracted ion chromatogram (EIC) of *m/z* 593.15 detected in negative ion detection mode

**Figure S55.** Proposed fragmentation pathway of C64 (Vicenin-2)

C65, C70, C75, C77, C81, C87, C90, C95: Fisetinidol-(4a→8)-catechin or isomer

Fisetinidol-(4a→8)-catechin^a^ Fisetinidol-(4β→8)-catechin^a^

ent-fisetinidol(4β→6)-catechin^a^

(2R,2'R,3S,3'S,4S)-2,2'-Bis(3,4-dihydroxyphenyl)-3,3',4,4'-tetrahydro[4,6'-bi-2H-1-benzopyran]-3,3',5',7,7'-pentol

(2R,2'R,3R,3'R,4R)-2,2'-Bis(3,4-dihydroxyphenyl)-3,3',4,4'-tetrahydro[4,8'-bi-2H-1-benzopyran]-3,3',5',7,7'-pentol

(2S,2'R,3R,3'S,4S)-2,2'-Bis(3,4-dihydroxyphenyl)-3,3',4,4'-tetrahydro[4,8'-bi-2H-1-benzopyran]-3,3',5',7,7'-pentol

(2R,2'R,3R,3'S,4R)-2,2'-Bis(3,4-dihydroxyphenyl)-3,3',4,4'-tetrahydro[4,8'-bi-2H-1-benzopyran]-3,3',5',7,7'-pentol

(2R,2'R,3R,3'S,4R)-2,2'-Bis(3,4-dihydroxyphenyl)-3,3',4,4'-tetrahydro[4,6'-bi-2H-1-benzopyran]-3,3',5',7,7'-pentol

**Figure S56.** Extracted ion chromatogram (EIC) of *m/z* 561.14 detected in negative ion detection mode

**Figure S57.** Proposed fragmentation pathway of C65, C70, C75, C77, C81, C87, C90 or C95 (Fisetinidol-(4a→8)-catechin or isomer)

C66: Cistanoside E or isomer

Cistanoside E

**Figure S58.** Extracted ion chromatogram (EIC) of *m/z* 475.18 detected in negative ion detection mode

**Figure S59.** Proposed fragmentation pathway of C66 (Cistanoside E)

C68, C71, C83, C85: Cinchonain IIa or isomer

Cinchonain IIa^a^ Cinchonain IIb^a^

Kandelin A 1 Kandelin A 2

**Figure S60.** Extracted ion chromatogram (EIC) of *m/z* 739.17 detected in negative ion detection mode

**Figure S61.** Proposed fragmentation pathway of C68, C71, C83 or C85 (Cinchonain IIa or isomer)

C74: 5*-O-*(6'*-O-*Galloyl*-β-D*-glucopyranosyl)gentisic acid or its isomer

5*-O-*(6'*-O-*Galloyl*-*β*-*D-glucopyranosyl)gentisic acid^a^

**Figure S62.** Extracted ion chromatogram (EIC) of *m/z* 467.08 detected in negative ion detection mode

**Figure S63.** Proposed fragmentation pathway of C74 (5*-O-*(6'*-O-*Galloyl*-*β*-*D-glucopyranosyl)gentisic acid)

C80, C94, C98, C100: Epiguibourtinidol-(4a→8)-catechin or isomer

Epiguibourtinidol-(4a→8)-catechin^a^ Guibourtinidol-(4β→8)-catechin^a^

Guibourtinidol-(4a→8)-catechin^a^

(2S,2'R,3R,3'R,4R)-2'-(3,4-Dihydroxyphenyl)-3,3',4,4'-tetrahydro-2-(4-hydroxyphenyl)[4,8'-bi-2H-1-benzopyran]-3,3',5',7,7'-pentol

**Figure S64.** Extracted ion chromatogram (EIC) of *m/z* 545.14 detected in negative ion detection mode

**Figure S65.** Proposed fragmentation pathway of C80, C94, C98 or C100 (Epiguibourtinidol-(4a→8)-catechin or isomer)

C82: Kaempferol 3*-O-β-D*-glucopyranosyl-7*-O-α-L*-rhamnopyranoside

Kaempferol 3*-O-*β*-*D-glucopyranosyl-7*-O-*α*-*L-rhamnopyranoside^a^

**Figure S66.** Extracted ion chromatogram (EIC) of *m/z* 593.15 detected in negative ion detection mode

**Figure S67.** Proposed fragmentation pathway of C82 (Kaempferol 3*-O-*β*-*D-glucopyranosyl-7*-O-*α*-*L-rhamnopyranoside)

C84: (2E,4E)-8-(β-D-Glucopyranosyloxy)-2,7-dimethyl-2,4-decadienedioic acid or isomer

(2E,4E)-8-(β*-*D-Glucopyranosyloxy)-2,7-dimethyl-2,4-decadienedioic acid

**Figure S68.** Extracted ion chromatogram (EIC) of *m/z* 403.16 detected in negative ion detection mode

**Figure S69.** Proposed fragmentation pathway of C84 ((2E,4E)-8-(β*-*D-Glucopyranosyloxy)-2,7-dimethyl-2,4-decadienedioic acid)

C91, C108, C111, C126, C132, C133: Cinchonain Ia or isomer

Cinchonain Ia^a^ Cinchonain Ib^a^

Rhinchoin Ia^a^ Cinchonain Ic

Cinchonain Cinchonain Id

**Figure S70.** Extracted ion chromatogram (EIC) of *m/z* 451.10 detected in negative ion detection mode

**Figure S71.** Proposed fragmentation pathway of C91, C108, C111, C126, C132 or C133 (Cinchonain Ia or isomer)

C92: Ellagic acid

Ellagic acid^a^

**Figure S72.** Extracted ion chromatogram (EIC) of *m/z* 301.00 detected in negative ion detection mode

**Figure S73.** Proposed fragmentation pathway of C92 (Ellagic acid)

C93, C97, C103, C107: Neoastilbin , Astilbin, Neoisoastilbin, Isoastilbin

Neoastilbin^a^ Astilbin^a^

Neoisoastilbin^a^ Isoastilbin^a^

**Figure S74.** Extracted ion chromatogram (EIC) of *m/z* 449.11 detected in negative ion detection mode

**Figure S75.** Proposed fragmentation pathway of C97 (Astilbin or isomer)

C96: Rutin

Rutin^a^

**Figure S76.** Extracted ion chromatogram (EIC) of *m/z* 609.14 detected in negative ion detection mode

**Figure S77.** Proposed fragmentation pathway of C96 (Rutin)

C99: Isoquercetin

Isoquercetin^a^

**Figure S78.** Extracted ion chromatogram (EIC) of *m/z* 463.09 detected in negative ion detection mode

**Figure S79.** Proposed fragmentation pathway of C99 (Isoquercetin)

C102: Acacetin 7*-O-*(6''*-O-*a-L-rhamnopyranosyl)*-*β*-*sophoroside

Acacetin 7*-O-*(6''*-O-*a-L-rhamnopyranosyl)*-*β*-*sophoroside^a^

**Figure S80.** Extracted ion chromatogram (EIC) of *m/z* 739.21 detected in negative ion detection mode

**Figure S81.** Proposed fragmentation pathway of C102 (Acacetin 7*-O-*(6''*-O-*a-L-rhamnopyranosyl)*-*β*-*sophoroside)

C104: Mikwelianin

Mikwelianin^a^

**Figure S82.** Extracted ion chromatogram (EIC) of *m/z* 477.07 detected in negative ion detection mode

**Figure S83.** Proposed fragmentation pathway of C104 (Mikwelianin)

C105: Aromadendrin

Aromadendrin^a^

**Figure S84.** Extracted ion chromatogram (EIC) of Aromadendrin detected in negative ion detection mode

**Figure S85.** Proposed fragmentation pathway of C105 (Dihydrokaempferol)

C106: Kaempferitrin

Kaempferitrin^a^

**Figure S86.** Extracted ion chromatogram (EIC) of *m/z* 577.14 detected in negative ion detection mode

**Figure S87.** Proposed fragmentation pathway of C106 (Kaempferitrin)

C109, C113, C114: Kaempferol-5*-O-*β-D-glucopyranoside, Kaempferol-7*-O-*β-D-glucopyranoside, Kaemferol-3*-O-*β-D-glucopyranoside

C117: Quercetin 3*-O-*a-L-rhamnopyranoside

Kaempferol-5*-O-*β-D-glucopyranoside^a^ Kaempferol-7*-O-*β-D-glucopyranoside^a^

Kaemferol-3*-O-*β-D-glucopyranoside^a^ Quercetin 3*-O-*a-L-rhamnopyranoside^a^

**Figure S88.** Extracted ion chromatogram (EIC) of *m/z* 447.09 detected in negative ion detection mode

**Figure S89.** Proposed fragmentation pathway of C109, C113 or C114 (Kaempferol-3*-O-*β-D-glucopyranoside or isomer)

**Figure S90.** Proposed fragmentation pathway of C117 (Quercetin-3*-O-*a-L-rhamnopyranoside)

C110: Nicotflorin

Nicotflorin^a^

**Figure S91.** Extracted ion chromatogram (EIC) of *m/z* 593.15 detected in negative ion detection mode

**Figure S92.** Proposed fragmentation pathway of C110 (Nicotflorin)

C116, C120, C123, C129: Isoengeletin, Engeletin, Neoisoengeletin, Neoengeletin

Isoengeletin^a^ Engeletin^a^

Neoisoengeletin Neoengeletin

**Figure S93.** Extracted ion chromatogram (EIC) of *m/z* 433.11 detected in negative ion detection mode

**Figure S94.** Proposed fragmentation pathway of C120 (Engeletin or isomer)

C121: Isorhamnetin-3*-O-*β-D-glucoside

Isorhamnetin-3*-O-*β-D-glucoside^a^

**Figure S95.** Extracted ion chromatogram (EIC) of *m/z* 477.10 detected in negative ion detection mode

**Figure S96.** Proposed fragmentation pathway of C121 (Isorhamnetin-3*-O-*β*-*D-glucoside)

C122: kaempferol-3*-O-*β-D-glucuronide

kaempferol-3*-O-*β-D-glucuronide^a^

**Figure S97.** Extracted ion chromatogram (EIC) of *m/z* 461.07 detected in negative ion detection mode

**Figure S98.** Proposed fragmentation pathway of C122 (Kaempferol-3*-O-*β*-*D-glucuronide)

C124, C127: 1-Methoxyoxalyl-3,5-dicaffeoylquinic acid or isomer

1-Methoxyoxalyl-3,5-dicaffeoylquinic acid

1-[(1α,2R,4α,6R)-4-Carboxy-2,6-bis[[3-(3,4-dihydroxyphenyl)-1-oxo-2-propen-1-yl]oxy]-4-hydroxycyclohexyl] hydrogen propanedioate

**Figure S99.** Extracted ion chromatogram (EIC) of *m/z* 601.12 detected in negative ion detection mode

**Figure S100.** Proposed fragmentation pathway of C124 or C127 (1-Methoxyoxalyl-3,5-dicaffeoylquinic acid or isomer)

C130: Afzelin

Afzelin^a^

**Figure S101.** Extracted ion chromatogram (EIC) of *m/z* 431.10 detected in negative ion detection mode

**Figure S102.** Proposed fragmentation pathway of C130 (Afzelin)

C131, C137, C149: Erigoster A or its isomers

Erigoster A

β-​lyxo-​2-​Octulopyranosonic acid, 2,​7-​anhydro-​3-​deoxy-​, methyl ester, 4,​8-​bis[(2E)​-​3-​(3,​4-​dihydroxyphenyl)​-​2-​propenoate]​, (7ξ)​-

β-​manno-​2-​Octulopyranosonic acid, 2,​7-​anhydro-​3-​deoxy-​, methyl ester, 4,​8-​bis[(2E)​-​3-​(3,​4-​dihydroxyphenyl)​-​2-​propenoate]

**Figure S103.** Extracted ion chromatogram (EIC) of *m/z* 557.13 detected in negative ion detection mode

**Figure S104.** Proposed fragmentation pathway of C131, C137 or C149 (Erigoster A or isomer)

C134, C135, C136, C138, C144, C150, C153, C155, C166, C169, C171, C173: (2α,3β,4β,19α)-23-(glycero-manno-Heptonoyloxy)-2,3,19 trihydroxyolean-12-en-28-oic acid or isomer

(2α,3β,4β,19α)-23-(glycero-manno-Heptonoyloxy)-2,3,19 trihydroxyolean-12-en-28-oic acid

(2α,3β,4α,6β)-23-(glycero-manno-Heptonoyloxy)-2,3,6-trihydroxyurs-12-en-28-oic acid

**Figure S105.** Extracted ion chromatogram (EIC) of *m/z* 711.40 detected in negative ion detection mode

**Figure S106.** Proposed fragmentation pathway of C134, C135, C136, C138, C144, C150, C153, C155, C166, C169, C171 or C173 ( (2α,3β,4β,19α)-23-(glycero-manno-Heptonoyloxy)-2,3,19 trihydroxyolean-12-en-28-oic acid or isomer)

C139: Quercetin

Quercetin^a^

**Figure S107.** Extracted ion chromatogram (EIC) of *m/z* 301.04 detected in negative ion detection mode

**Figure S108.** Proposed fragmentation pathway of C139 (Quercetin)

C140, C141: Madecassoside, Asiaticoside B

Madecassoside^a^ Asiaticoside B^a^

**Figure S109.** Extracted ion chromatogram (EIC) of *m/z* 973.50 detected in negative ion detection mode

**Figure S110.** Proposed fragmentation pathway of C140 (Madecassoside or isomer)

C142, C147: Smilaside B or isomer

Smilaside B^a^ Helonioside B

**Figure S111.** Extracted ion chromatogram (EIC) of *m/z* 735.21 detected in negative ion detection mode

**Figure S112.** Proposed fragmentation pathway of C142 or C147 (Smilaside B or isomer)

C143, C145, C146, C148, C154, C159, C162, C163, C165, C170, C172:

Asiaticoside or isomer

Asiaticoside^a^ Centellasaponin D^a^ Centellasaponin C^a^

(2a,3β,6β)-trihydroxyolean-12-en-28-oic acid O-a-L-rhamnopyranosyl-(1→4)*-O-*β-D-glucopyranosyl-(1→6)*-O-*β-D-glucopyranosy ester^a^

Centellasaponin A^a^ Isoasiaticoside^a^ Scheffoleoside A^a^

Scheffursoside F^a^ Scheffoleoside F Centellasaponin J^a^

(3β,6β,23)-trihydroxyurs-12-en-28-oic acid O-a-L-rhamnopyranosyl-(1→4)*-O-*β-D-glucopyranosyl-(1→6)*-O-*β-D-glucopyranosy ester^a^

**Figure S113.** Extracted ion chromatogram (EIC) of *m/z* 957.51 detected in negative ion detection mode

**Figure S114.** Extracted ion chromatogram (EIC) of *m/z* 1003.50 detected in negative ion detection mode

**Figure S115.** Proposed fragmentation pathway of C148 (Asiaticoside or isomer)

C151, C152, C156, C158, C161, C168: Smilaside A or isomer

Smilaside A^a^ 2',6'-Diacetyl-3,6-diferuloylsucrose

Smilaside M Smilaside N

α-D-Glucopyranoside, 3,6-bis*-O-*[(2E)-3-(4-hydroxy-3-methoxyphenyl)-1-oxo-2-propenyl]-β-D-fructofuranosyl, 3,6-diacetate (9CI)

α-​D-​Glucopyranoside, 1-​O-​acetyl-​3,​6-​bis-​O-​[(2E)​-​3-​(4-​hydroxy-​3-​methoxyphenyl)​-​1-​oxo-​2-​propen-​1-​yl]​-​β-​D-​fructofuranosyl, 2-​acetate

**Figure S116.** Extracted ion chromatogram (EIC) of *m/z* 777.22 detected in negative ion detection mode

**Figure S117.** Proposed fragmentation pathway of C151, C152, C156, C158, C161 or C168 (Smilaside A or isomer)

C157: Kaempferol

Kaempferol^a^

**Figure S118.** Extracted ion chromatogram (EIC) of *m/z* 285.10 detected in negative ion detection mode

**Figure S119.** Proposed fragmentation pathway of C157 (Kaempferol)

C160, C174, C175, C177: Madecassic acid or isomer

C179, C180: Camelliagenin E or isomer

Madecassic acid^a^ Terminolic acid^a^ 19α-Hydroxyasiatic acid^a^

Isothankunic acid^a^

Camelliagenin E Camelliagenin D

**Figure S120.** Extracted ion chromatogram (EIC) of *m/z* 503.34 detected in negative ion detection mode

**Figure S121.** Proposed fragmentation pathway of C160 (Madecassic acid isomer)

**Figure S122.** Proposed fragmentation pathway of C174, C175 or C177 (Madecassic acid or isomer)

**Figure S123.** Proposed fragmentation pathway of C179 or C180 (Camelliagenin E or isomer)

C164: 3-[5'-(2-Carboxyethenyl)-2',6-dihydroxy-3',5-dimethoxy[1,1'-biphenyl]-3-yl]-2-[4-(2-carboxyethenyl)-2-methoxyphenoxy]-2-propenoic acid

3-[5'-(2-Carboxyethenyl)-2',6-dihydroxy-3',5-dimethoxy[1,1'-biphenyl]-3-yl]-2-[4-(2-carboxyethenyl)-2-methoxyphenoxy]-2-propenoic acid

**Figure S124.** Extracted ion chromatogram (EIC) of *m/z* 577.14 detected in negative ion detection mode

**Figure S125.** Proposed fragmentation pathway of C164 ((3-[5'-(2-Carboxyethenyl)-2',6-dihydroxy-3',5-dimethoxy[1,1'-biphenyl]-3-yl]-2-[4-(2-carboxyethenyl)-2-methoxyphenoxy]-2-propenoic acid)

C167: 9,12,13-trihydroxy-octadecenoic acid

9,12,13-trihydroxy-octadecenoic acid^a^

**Figure S126.** Extracted ion chromatogram (EIC) of *m/z* 331.25 detected in negative ion detection mode

**Figure S127.** Proposed fragmentation pathway of C167 (9,12,13-trihydroxy-octadecenoic acid)

C176, C178: 24-​Noroleana-​3,​12-​dien-​28-​oic acid, 3,​15-​dihydroxy-​2-​oxo-​, β-​D-​glucopyranosyl ester or isomer

24-​Noroleana-​3,​12-​dien-​28-​oic acid, 3,​15-​dihydroxy-​2-​oxo-​, β-​D-​glucopyranosyl ester

**Figure S128.** Extracted ion chromatogram (EIC) of *m/z* 677.35 detected in negative ion detection mode

**Figure S129.** Proposed fragmentation pathway of C176 or C178 (24-Noroleana-3,12-dien-28-oic acid, 3,15-dihydroxy-2-oxo-, β-D-glucopyranosyl ester or isomer)

C181, C182, C183, C184, C185: Alpinoside or isomer

Alpinoside^a^ Rubuside B^a^

Glycyrrhetinic acid-3*-O-*monoglucose

β-D-Glucopyranosyl (3β,20β)-3-hydroxy-11-oxoolean-12-en-29-oate

β-D-Glucopyranosyl (3β,4α)-3,23-dihydroxyursa-12,18-dien-28-oate

**Figure S130.** Extracted ion chromatogram (EIC) of *m/z* 677.39 detected in negative ion detection mode

**Figure S131.** Proposed fragmentation pathway of C181, C182, C183, C184 or C185 (Alpinoside or isomer)

C186, C189, C192, C193: Tormentic acid or isomer

Tormentic acid^a^ Arjunolic acid^a^ Uncargenin C^a^

Euscaphic acid^a^

**Figure S132.** Extracted ion chromatogram (EIC) of *m/z* 487.34 detected in negative ion detection mode

**Figure S133.** Proposed fragmentation pathway of C186, C189, C192 or C193 (Tormentic acid or isomer)

C187, C188: Esculentoside B or isomer

Esculentoside B Dianoside F

**Figure S134.** Extracted ion chromatogram (EIC) of *m/z* 663.37 detected in negative ion detection mode

**Figure S135.** Proposed fragmentation pathway of C187 or C188 (Esculentoside B or isomer)

C190, C191, C194, C195, C196: Fupenzic acid or isomer

Fupenzic acid^a^  (15α)-15,19-Dihydroxy-3-oxoursa-1,12-dien-28-oic acid

19α-Hydroxy-3,6-dioxours-12-en-28-oic acid 11-Oxo-pomonic acid

3-Oxo-15α-hydroxyglycyrrhetinic acid

**Figure S136.** Extracted ion chromatogram (EIC) of *m/z* 483.31 detected in negative ion detection mode

**Figure S137.** Proposed fragmentation pathway of C190, C191, C194, C195 or C196 (Fupenzic acid or isomer)

# Mass spectrometry data of 44 compound references

C1: Malic acid

**Figure S138.** Extracted ion chromatogram (EIC) of Malic acid detected in negative ion detection mode

**Figure S139.** (MS^n^) spectra of Malic acid detected in negative ion detection mode

C7: Gallic acid

**Figure S140.** Extracted ion chromatogram (EIC) of Gallic acid detected in negative ion detection mode

**Figure S141.** (MS^n^) spectra of Gallic acid detected in negative ion detection mode

C19 Neochlorogenic acid

**Figure S142.** Extracted ion chromatogram (EIC) of Neochlorogenic acid detected in negative ion detection mode

**Figure S143.** (MS^n^) spectra of Neochlorogenic acid detected in negative ion detection mode

C27: Procyanidin B3

**Figure S144.** Extracted ion chromatogram (EIC) of Procyanidin B3 detected in negative ion detection mode

**Figure S145.** (MS^n^) spectra of Procyanidin B3 detected in negative ion detection mode

C31: (+)-Catechin

**Figure S146.** Extracted ion chromatogram (EIC) of (+)-Catechin detected in negative ion detection mode

**Figure S147.** (MS^n^) spectra of (+)-Catechin detected in negative ion detection mode

C35: Caffeic acid

**Figure S148.** Extracted ion chromatogram (EIC) of Caffeic acid detected in negative ion detection mode

**Figure S149.** (MS^n^) spectra of Caffeic acid detected in negative ion detection mode

C37: Chlorogenic acid

**Figure S150.** Extracted ion chromatogram (EIC) of Chlorogenic acid detected in negative ion detection mode

**Figure S151.** (MS^n^) spectra of Chlorogenic acid detected in negative ion detection mode

C41: Cryptochlorogenic acid

**Figure S152.** Extracted ion chromatogram (EIC) of Cryptochlorogenic acid detected in negative ion detection mode

**Figure S153.** (MS^n^) spectra of Cryptochlorogenic acid detected in negative ion detection mode

C52: (-)-Epicatechin

**Figure S154.** Extracted ion chromatogram (EIC) of (-)-Epicatechin detected in negative ion detection mode

**Figure S155.** (MS^n^) spectra of (-)-Epicatechin detected in negative ion detection mode

C59: 5*-O-*caffeoylshikimic acid

**Figure S156.** Extracted ion chromatogram (EIC) of 5*-O-*caffeoylshikimic acid detected in negative ion detection mode

**Figure S157.** (MS^n^) spectra of 5*-O-*caffeoylshikimic acid detected in negative ion detection mode

C61: 1,3-Dicaffeoylquinic acid

**Figure S158.** Extracted ion chromatogram (EIC) of 1,3-Dicaffeoylquinic acid detected in negative ion detection mode

**Figure S159.** (MS^n^) spectra of 1,3-Dicaffeoylquinic acid detected in negative ion detection mode

C64: Vicenin-2

**Figure S160.** Extracted ion chromatogram (EIC) of Vicenin-2 detected in negative ion detection mode

**Figure S161.** (MS^n^) spectra of Vicenin-2 detected in negative ion detection mode

C92: Ellagic acid

**Figure S162.** Extracted ion chromatogram (EIC) of Ellagic acid detected in negative ion detection mode

**Figure S163.** (MS^n^) spectra of Ellagic acid detected in negative ion detection mode

C93: Neoastilbin

**Figure S164.** Extracted ion chromatogram (EIC) of Neoastilbin detected in negative ion detection mode

**Figure S165.** (MS^n^) spectra of Neoastilbin detected in negative ion detection mode

C96: Rutin

**Figure S166.** Extracted ion chromatogram (EIC) of Rutin detected in negative ion detection mode

**Figure S167.** (MS^n^) spectra of Rutin detected in negative ion detection mode

C97: Astilbin

**Figure S168.** Extracted ion chromatogram (EIC) of Astilbin detected in negative ion detection mode

**Figure S169.** (MS^n^) spectra of Astilbin detected in negative ion detection mode

C99: Isoquercetin

**Figure S170.** Extracted ion chromatogram (EIC) of Isoquercetin detected in negative ion detection mode

**Figure S171.** (MS^n^) spectra of Isoquercetin detected in negative ion detection mode

C103: Neoisoastilbin

**Figure S172.** Extracted ion chromatogram (EIC) of Neoisoastilbin detected in negative ion detection mode

**Figure S173.** (MS^n^) spectra of Neoisoastilbin detected in negative ion detection mode

C104: Mikwelianin

**Figure S174.** Extracted ion chromatogram (EIC) of Mikwelianin detected in negative ion detection mode

**Figure S175.** (MS^n^) spectra of Mikwelianin detected in negative ion detection mode

C105: Aromadendrin

**Figure S176.** Extracted ion chromatogram (EIC) of Aromadendrin detected in negative ion detection mode

**Figure S177.** (MS^n^) spectra of Aromadendrin detected in negative ion detection mode

C106: Kaempferitrin

**Figure S178.** Extracted ion chromatogram (EIC) of Kaempferitrin detected in negative ion detection mode

**Figure S179.** (MS^n^) spectra of Kaempferitrin detected in negative ion detection mode

C107: Isoastilbin

**Figure S180.** Extracted ion chromatogram (EIC) of Isoastilbin detected in negative ion detection mode

**Figure S181.** (MS^n^) spectra of Isoastilbin detected in negative ion detection mode

C110: Nicotflorin

**Figure S182.** Extracted ion chromatogram (EIC) of Nicotflorin detected in negative ion detection mode

**Figure S183.** (MS^n^) spectra of Nicotflorin detected in negative ion detection mode

C112: Isochlorogenic acid B

**Figure S184.** Extracted ion chromatogram (EIC) of Isochlorogenic acid B detected in negative ion detection mode

**Figure S185.** (MS^n^) spectra of Isochlorogenic acid B detected in negative ion detection mode

C113: Kaemferol-7*-O-*β-D-glucopyranoside

**Figure S186.** Extracted ion chromatogram (EIC) of Kaemferol-7*-O-*β-D-glucopyranoside detected in negative ion detection mode

**Figure S187.** (MS^n^) spectra of Kaemferol-7*-O-*β-D-glucopyranoside detected in negative ion detection mode

C114: Kaemferol-3*-O-*β-D-glucopyranoside

**Figure S188.** Extracted ion chromatogram (EIC) of Kaemferol-3*-O-*β-D-glucopyranoside detected in negative ion detection mode

**Figure S189.** (MS^n^) spectra of Kaemferol-3*-O-*β-D-glucopyranoside detected in negative ion detection mode

C115: Isochlorogenic acid A

**Figure S190.** Extracted ion chromatogram (EIC) of Isochlorogenic acid A detected in negative ion detection mode

**Figure S191.** (MS^n^) spectra of Isochlorogenic acid A detected in negative ion detection mode

C117: Quercetin 3*-O-*a-L-rhamnopyranoside

**Figure S192.** Extracted ion chromatogram (EIC) of Quercetin 3*-O-*a-L-rhamnopyranoside detected in negative ion detection mode

**Figure S193.** (MS^n^) spectra of Quercetin 3*-O-*a-L-rhamnopyranoside detected in negative ion detection mode

C118: 1,4-Dicaffeoylquinic acid

**Figure S194.** Extracted ion chromatogram (EIC) of 1,4-Dicaffeoylquinic acid detected in negative ion detection mode

**Figure S195.** (MS^n^) spectra of 1,4-Dicaffeoylquinic acid detected in negative ion detection mode

C119: 1,5-Dicaffeoylquinic acid

**Figure S196.** Extracted ion chromatogram (EIC) of 1,5-Dicaffeoylquinic acid detected in negative ion detection mode

**Figure S197.** (MS^n^) spectra of 1,5-Dicaffeoylquinic acid detected in negative ion detection mode

C120: Engeletin

**Figure S198.** Extracted ion chromatogram (EIC) of Engeletin detected in negative ion detection mode

**Figure** **S199.** (MS^n^) spectra of Engeletin detected in negative ion detection mode

C121: Isorhamnetin-3*-O-*β-D-glucoside

**Figure S200.** Extracted ion chromatogram (EIC) of Isorhamnetin-3*-O-*β-D-glucoside detected in negative ion detection mode

**Figure S201.** (MS^n^) spectra of Isorhamnetin-3*-O-*β-D-glucoside detected in negative ion detection mode

C125: Phloridzin

**Figure S202.** Extracted ion chromatogram (EIC) of Phloridzin detected in negative ion detection mode

**Figure S203.** (MS^n^) spectra of Phloridzin detected in negative ion detection mode

C128: Isochlorogenic acid C

**Figure S204.** Extracted ion chromatogram (EIC) of Isochlorogenic acid C detected in negative ion detection mode

**Figure S205.** (MS^n^) spectra of Isochlorogenic acid C detected in negative ion detection mode

C130: Afzelin

**Figure S206.** Extracted ion chromatogram (EIC) of Afzelin detected in negative ion detection mode

**Figure S207.** (MS^n^) spectra of Afzelin detected in negative ion detection mode

C139: Quercetin

**Figure S208.** Extracted ion chromatogram (EIC) of Quercetin detected in negative ion detection mode

**Figure S209.** (MS^n^) spectra of Quercetin detected in negative ion detection mode

C140: Madecassoside

**Figure S210.** Extracted ion chromatogram (EIC) of Madecassoside detected in negative ion detection mode

**Figure S211.** (MS^n^) spectra of Madecassoside detected in negative ion detection mode

C140: Asiaticoside B

**Figure S212.** Extracted ion chromatogram (EIC) of Asiaticoside B detected in negative ion detection mode

**Figure S213.** (MS^n^) spectra of Asiaticoside B detected in negative ion detection mode

C148: Asiaticoside

**Figure S214.** Extracted ion chromatogram (EIC) of Asiaticoside detected in negative ion detection mode

**Figure S215.** (MS^n^) spectra of Asiaticoside detected in negative ion detection mode

C157: Kaempferol

**Figure S216.** Extracted ion chromatogram (EIC) of Kaempferol detected in negative ion detection mode

**Figure S217.** (MS^n^) spectra of Kaempferol detected in negative ion detection mode

C174: 19α-Hydroxyasiatic acid

**Figure S218.** Extracted ion chromatogram (EIC) of 19α-Hydroxyasiatic acid detected in negative ion detection mode

**Figure S219.** (MS^n^) spectra of 19α-Hydroxyasiatic acid detected in negative ion detection mode

C177: Madecassic acid

**Figure S220.** Extracted ion chromatogram (EIC) of Madecassic acid detected in negative ion detection mode

**Figure S221.** (MS^n^) spectra of Madecassic acid detected in negative ion detection mode

C192: Tormentic acid

**Figure S222.** Extracted ion chromatogram (EIC) of Tormentic acid detected in negative ion detection mode

**Figure S223.** (MS^n^) spectra of Tormentic acid detected in negative ion detection mode

C193: Euscaphic acid

**Figure S224.** Extracted ion chromatogram (EIC) of Euscaphic acid detected in negative ion detection mode

**Figure S225.** (MS^n^) spectra of Euscaphic acid detected in negative ion detection mode
